# Community dynamics and metagenomic analyses reveal *Bacteroidota's* role in widespread enzymatic *Fucus vesiculosus* cell wall degradation

**DOI:** 10.1038/s41598-024-60978-8

**Published:** 2024-05-03

**Authors:** Jascha F. H. Macdonald, Pablo Pérez-García, Yannik K.-H. Schneider, Patrick Blümke, Daniela Indenbirken, Jeanette H. Andersen, Ines Krohn, Wolfgang R. Streit

**Affiliations:** 1 Department of Microbiology and Biotechnology, Biocenter Klein Flottbek, Institute of Plant Science and Microbiology, University of Hamburg, Ohnhorststr.18, 22609 Hamburg, Germany; 2 Institute for General Microbiology, Molecular Microbiology, Kiel University, Kiel, Germany; 3 Marbio, Faculty of Biosciences, Fisheries and Economics, UiT - The Arctic University of Norway, Tromsø, Norway; 4 Technology Platform Next Generation Sequencing, Leibniz Institute of Virology, Hamburg, Germany

**Keywords:** *Fucus vesiculosus*, Cell wall degradation, *Bacteroidota*, α-L-fucosidases, Microbiome, Microbial ecology, Bacterial genes

## Abstract

Enzymatic degradation of algae cell wall carbohydrates by microorganisms is under increasing investigation as marine organic matter gains more value as a sustainable resource. The fate of carbon in the marine ecosystem is in part driven by these degradation processes. In this study, we observe the microbiome dynamics of the macroalga *Fucus vesiculosus* in 25-day-enrichment cultures resulting in partial degradation of the brown algae. Microbial community analyses revealed the phylum *Pseudomonadota* as the main bacterial fraction dominated by the genera *Marinomonas* and *Vibrio*. More importantly, a metagenome-based Hidden Markov model for specific glycosyl hydrolyses and sulphatases identified *Bacteroidota* as the phylum with the highest potential for cell wall degradation, contrary to their low abundance. For experimental verification, we cloned, expressed, and biochemically characterised two α-L-fucosidases, FUJM18 and FUJM20. While protein structure predictions suggest the highest similarity to a *Bacillota* origin, protein–protein blasts solely showed weak similarities to defined *Bacteroidota* proteins. Both enzymes were remarkably active at elevated temperatures and are the basis for a potential synthetic enzyme cocktail for large-scale algal destruction.

## Introduction

Enzymatic algae cell wall carbohydrate (ACW) hydrolysis is a complex process with increasing attention in both industrial and ecological spheres. It enables the use of marine and freshwater algae as sustainable resources for valuable biomolecules already cultivated in large-scale algae farms^1^. So far, ACW carbohydrates are degraded in digestion tanks by microorganisms with relatively expensive conversion methods. Increasing the efficiency of the degrading communities and enzymes could benefit multiple fields of applications, such as the renewable energy sector in biofuel production and pharma and nutraceutical industries^2–5^.

Moreover, marine algae play a significant role in global carbon fluxes within natural marine systems. Understanding multitrophic community interactions and the primary drivers of enzymatic activities associated with the fate of carbon could help to elucidate fundamental processes in marine biogeochemistry^6^. Combining ecological and molecular methods results in new knowledge and insights into ecosystem health for conserving natural systems and understanding their response to global climate change.

So far, the cell disruption of ACWs lacks a needed, universal, and efficient industrial standard. Microbial substrate degradation has a high potential for future sustainable uses. The high variability in size, shape, and bioactive substances within the 169,000 species documented in AlgaeBase (https://www.algaebase.org) poses a challenge in establishing an standard procedure for efficiently processing different kinds of algae^7^. However, screening for novel enzymes for specific uses and substrates is still under significant investigation. This search benefits from new molecular tools such as “omics”-based analyses and improved protein function prediction methods.

The schematic structure of ACWs mainly consists of a matrix and composition of specific polysaccharides and proteins with unique biochemical characteristics in *Phaeophyceae*, *Rhodophyta*, *Chlorophyta*, and *Bacillariophyceae* (*Diatoms*), which share biochemical similarities to brown algae. The cell wall of the macroalgae species *Fucus vesiculosus* is composed of the 3 *Phaeophycean* associated polysaccharides fucoidan, alginate, and cellulose. Additionally, laminarin serves as a storage carbohydrate in the cell^8–11^. The saccharide composition, as well as linkage variation within the polymer structure, differ inter- and intraspecific. It depends on several biotic and abiotic influences such as seasonality, illumination, temperature, nutrient and CO_2_-concentration, age, and part of the organism^12–17^. These influences lead to a high variability of fucoidan in algae, such as species from the genus *Fucus*. The polysaccharide shows differences in, e.g. the molecular weight of 7 to 1600 kDa, the sulphated content between 9 and 40%, and the L-fucose content ranging from 25 up to 93%^18–20^.

Consequently, a universal degradation by microorganisms presupposes variable and multiple enzyme compositions. Fucoidan-degrading marine bacteria, such as *Lentimonas* spp., have already been described as reflecting a variable degradation capability by providing hundreds of different fucoidan-targeting enzymes of several classes and families^21^. Similar findings were made in kelp forest macroalgae species of the genus *Laminaria* with a major but varying carbohydrate content of laminarin, so findings from the present study can lead to solutions in multiple fields^22^. Since *F. vesiculosus* is easy to gather in nature in high quantities, it is suitable as a model organism. It is found in shelf regions all over the northern Atlantic. Furthermore, other *Fucus* species are distributed almost globally with high numbers in the northern Atlantic and Pacific. Therefore, brown algae cell wall polymers are under investigation in this study, focusing on fucoidan.

Similar to varying carbohydrate contents and structures in algae, marine microbial community composition is influenced by several biotic and abiotic factors such as seasonality, salinity, temperature, depth, or location, and is directly connected to substrate concentrations^23–25^. Previous phylogenetic studies have characterized the natural composition of bacterial species on *Fucus* surfaces in the Baltic Sea. The bacterial community mainly consists of *Pseudomonadota* species with a majority of *α*- and *γ*-*proteobacteria*^26–28^. Isolation approaches unveiled species within this phylum that have developed physiological adaptations to macroalgae surface areas and are genetically tailored to this habitat^29^. *Bacteroidota* comprises only around 1% of the bacterial community on previously investigated natural *Fucus* surface microbiomes^26,30^. However, these findings depend on the individual sampling dates, as *Bacteroidota* abundancies are influenced by seasonality^31^. In enrichment experiments over several days, *Bacteroidota* abundancies increased to 20–30%^27,32^. Furthermore, relatively high abundances of *Cyanobacteria* and *Planctomycetota* have been observed in investigations of natural Fucus microbiomes^33^.

Microbial degradation of ACW polymers achieves hydrolysis and cleavage of the glycosidic bond within the polysaccharide structure. This process separates crosslinked structures and cuts long polysaccharide chains into shorter fragments, enabling microorganisms to metabolize and transport the resulting sugar dimers or monomers. Additionally, various secreted enzymes modify the carbohydrate molecule structure.

These carbohydrate-cleaving and modifying enzymes are categorised in the Carbohydrate-Active enZYmes database (CAZy, http://www.cazy.org/) into enzyme function-defined classes and families like glycosidases (EC 3.2.1.-), esterase (EC 3.1.-), transferases (EC 2.4.-), lyases (EC 4.2.2.-), as well as associated modules^34^. Furthermore, the database Sulfatlas (https://sulfatlas.sb-roscoff.fr/) offers a classification of sulphatases (SU) that are active on sulphated biomolecules, such as the polysaccharide fucoidan in brown algae cell walls^35–37^.

**Figure 1:**
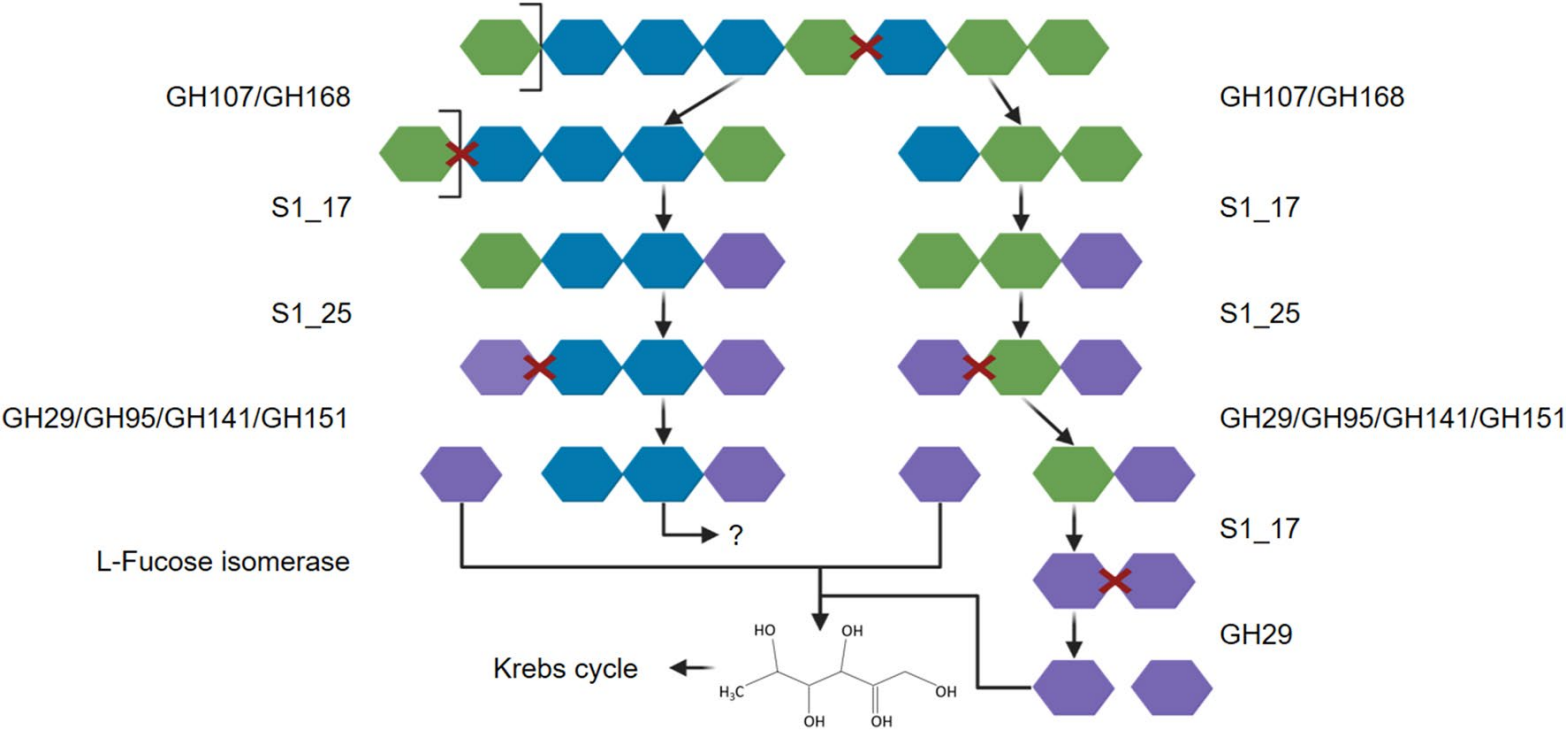
Schematic fucoidan degradation catalysis. Active enzymes from the GH and sulphatase families degrade the schematic structure of homofucan with sulphated α-1,3/1,4-linked L-fucose in multiple steps. Colour key: blue: 2 sulphate groups, green: 1 sulphate group, purple: L-fucose. Adapted from Li et al. (2022)^1^. Created with BioRender.com.

For our model organism *Fucus vesiculosus* and its main ACW carbohydrate fucoidan, the cascade of degradation reactions involves enzymes from the families GH29, GH95, GH107, GH141, GH151, GH168, as well as sulphatases S1_17 and S1_25 (Fig. 1)^21,38–40^. Significantly, family GH29 α-L-fucosidases that target four different linkage types (EC 2.3.1.51; EC 3.2.1.111; EC 3.2.63; EC 3.2.1.127) is of major interest and is involved in the hydrolysis steps resulting in L-fucose monomers.

Numerous studies focus on the polysaccharide degradation potential of *Bacteroidota*. The process of expressing corresponding genes, the secretion system and the carbohydrate transport of this phylum is complex^41^. *Bacteroidota* species offer hundreds of polysaccharide utilisation loci to regulate access to complex carbohydrates and prove their glycan-degrading capabilities^42–44^. Furthermore, starch utilisation systems and TonB-transporters are involved in transporting oligomers into the cells^45,46^.

Our study combines in vitro enrichment culture approaches with metagenomic and in silico approaches to enable insights into the degrading community regardless of isolation obstacles. Therefore, the so-called “viable but nonculturable” bacteria and their genes remain no longer unknown^47^. We want to evaluate the community dynamics and the enzymatic algae degradation potential in enrichment cultures using “omics”-based analyses. Our observations should allow insights into enrichment advantages and challenges for future algae degradation investigations. Furthermore, they facilitate the screening for novel putative fucoidan degrading enzymes to address the lack of established hydrolysing proteins targeting ACW carbohydrates. Additionally, we want to use our approach to identify and confirm new GH29 enzymes for fucoidan degradation.

## Results

### Initial Fucus-degradation potential of microbial communities

We observe the impact of microbial degradation in enrichment cultures by multiple metabolic approaches. Our initial testing suggested that the naturally attached microbial community of *Fucus vesiculosus* has great potential for degrading algae. Artificial inoculations with microbiomes from different origins, like soils or animal faeces, did not show comparable effects of biological activity (unpublished data). In the final experimental approach, we utilised 500 ml lightly sealed Schott-flasks for enrichment cultures. These cultures included 300 ml synthetic seawater (Tropic Marin Classic Meersalz, Hünenberg, Switzerland) and 50 g *F. vesiculosus* collected at Kiel Bight, Germany. We shook the flasks at 60 rpm in a 20 °C environment and adjusted the salinity to 15.

**Figure 2:**
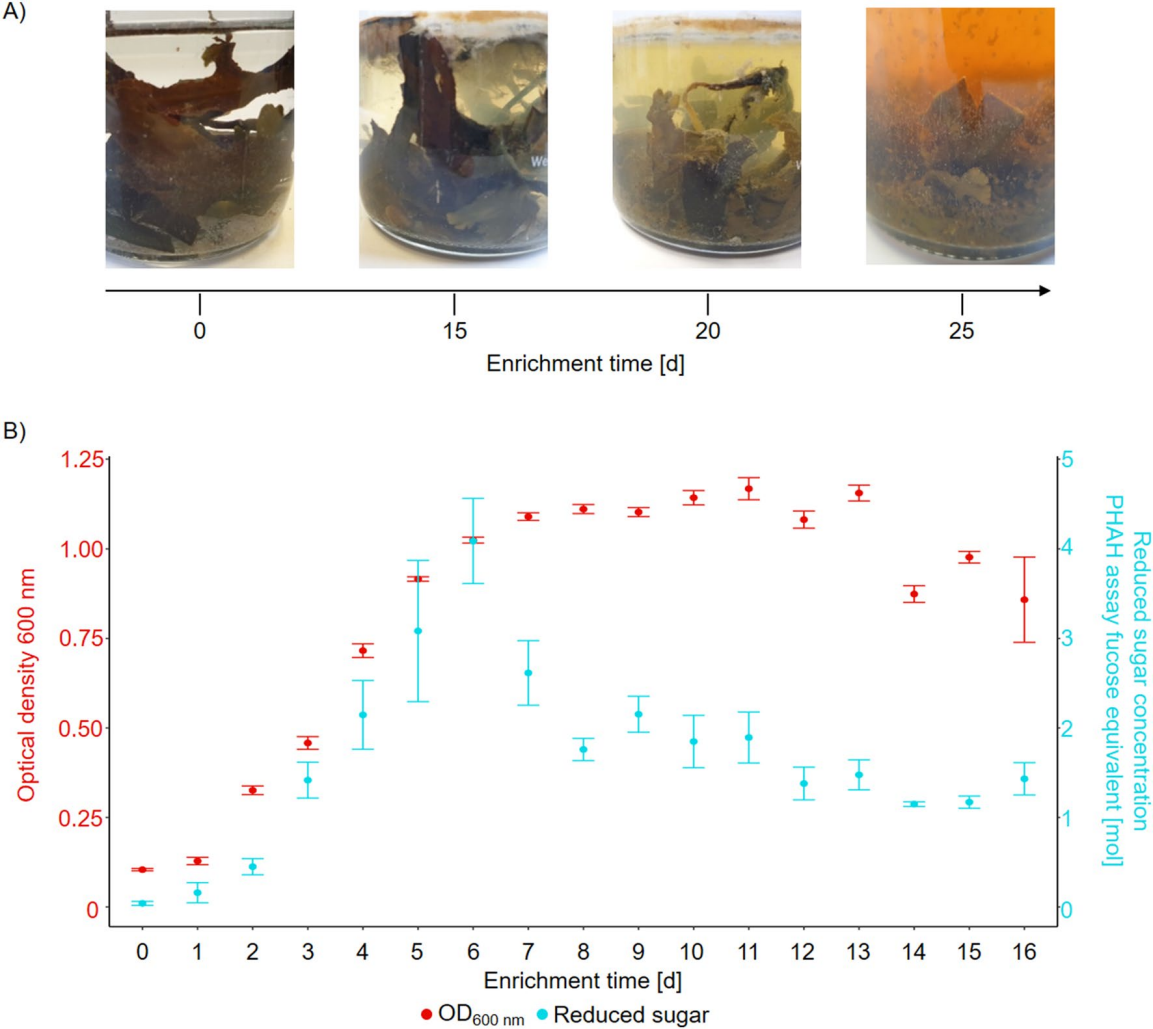
*Fucus vesiculosus* enrichment culture with naturally attached microbiome in synthetic seawater medium. (**A**) Visual changes in the algae structure medium turbidity and colour, as well as microbial growth and biofilm formation on the surface. (**B**) Quantification of microbial degradation of *F. vesiculosus* in enrichment cultures over 16 days in triplicates. Colour key: Red dots/left y-axis: optical density of the enrichment culture supernatant at 600 nm. Blue dots/right y-axis: reduced sugar concentration determined with the *para*-hydroxybenzoic acid assay. A standard curve with glucose as an equivalent was used to estimate the sugar concentration in relation.

Visual observation of *F. vesiculosus* enrichment culture with a naturally attached microbiome showed a significant change in the plant’s structure during the enrichment. A healthy *F. vesiculosus* appearance changed to a mush of plant fractions and cell debris (Fig. 2A). The release of plant cell materials and fractionated alga and the growth of different microorganisms led to a change in the colour of the enrichment medium from clear to greenish to brownish. Furthermore, a smell of sulphur increased, indicating a potential release of thiol groups in organosulphur compounds. After the displayed 25 days (d), the activity in the enrichment cultures comes to a stop with the red-brown colour and less turbidity. The overall weight loss of 50 g *F. vesiculosus* over 20 days in the enrichment set-up was 10.76 ± 0.07 g (21.52 ± 0.14%, n = 3). The *para*-hydroxybenzoic acid hydrazide (*p*hah) assay revealed an increase in the concentration of reducing sugar equivalents starting at 0.04 ± 0.02 mol of fitted values to a L-fucose standard curve and peaks on day 6 (d6) at 4.09 ± 0.47 mol. The concentration decreased and fluctuated from d7 to d16 between 2.61 ± 0.36 mol and 1.15 ± 0.03 mol (Fig. 2B, Supplementary Table 1). The optical density (OD_600nm_) was monitored as an indicator of the visual change of the medium. The enrichment culture was shaken before sampling to create a heterogeneous distribution of biomass. Microbial density, biofilm formation, and the release of degradation products, such as demolished small algae parts, actively influenced the optical density. Due to the combination of these processes, the OD_600nm_ increased from d0-d7 and fluctuated around a level of 1 in the following.

Ultra High-Performance Liquid Chromatography-Electrospray Ionisation-High Resolution-Mass Spectrometry2 (UHPLC-ESI-HR-MS2) data was recorded to track the composition change throughout the enrichment cultures. Three parallel enrichment culture samples were taken and analysed after 1, 10 and 31 days. Principal component analysis (PCA) of the MS data was used to simplify the datasets (Supplementary Fig. 1). Negative electrospray ionisation (ESI-) was chosen beside positive electrospray ionization (ESI +), since we expected a high number of carbohydrate metabolites when degrading algae-biomass (Supplementary Fig. 1B). The hydroxyl and carboxyl groups of carbohydrates are not prone to protonation and appear in ESI- commonly as [M-H]^−^ while in ESI + molecules bearing functional groups prone to protonation such as amines enable the detection of positively charged ions and adducts ([M + H]^+^/[M + Na]^+^). We have chosen the additional metabolomic analysis to encompass a broad range of metabolites in order to track the change in metabolites between the replicates. Three replicates for each time point group together within the two-dimensional PCA for negative and positive ESI. A common change of the chemical composition over time was observed, between 1 and 10 days and 10 and 31 days for all replicates. This change hints at a similar molecular degradation process as indicated by the metabolites in the enrichment cultures. Further, the degradation of *F. vesiculosus* within the first 1–10 days differed from the degradation between 10 and 31 days. These results supported the findings of the *p*HAH assay and the sugar dynamics (Fig. 2B) and are also represented by the successful degradation process in the enrichment cultures (Fig. 2B).

### Bacterial community dynamics

**Figure 3:**
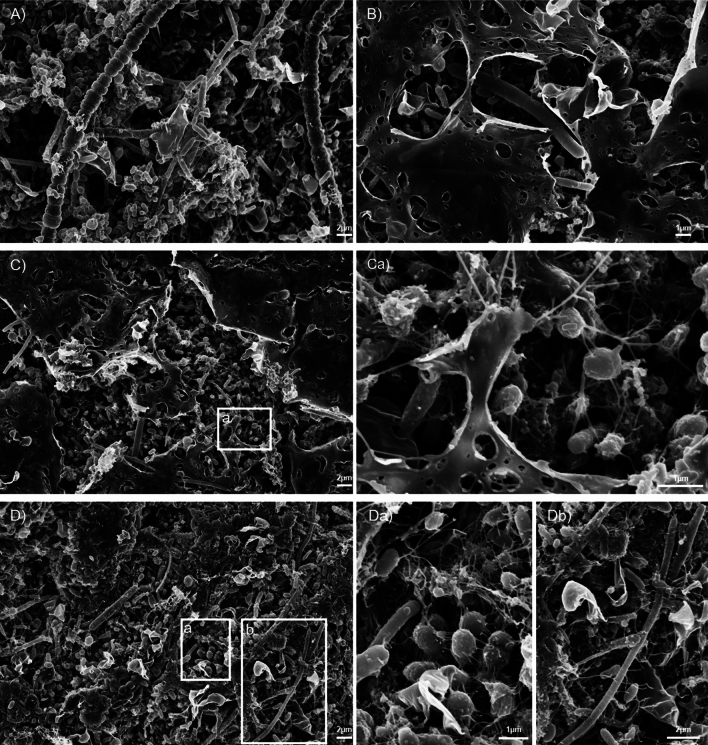
Scanning electron microscopy images of *F. vesiculosus* surfaces and attached microbiome in enrichment cultures after 9 days. (**A**) Microorganisms on degraded algae parts. Characteristic cell stacks are visible. (**B**) Degraded algae tissue. (**C**) Microbial clusters are concentrated in degraded algae parts with Ca) filamentous attachments of bacteria to the substrate and between individuals. (**D**) Visual diversity of bacteria, possible aggregates, and organic attachments with variations in shape and sizes in Da) and Db) between < 1 and > 15 µm. Scale bars are indicated in the images (Zeiss Leo Gemini 1525 FEG SEM; 5.00 kV).

The microbial community in *F. vesiculosus* enrichment cultures consisted of a diverse mix of microorganisms. SEM image analyses revealed insights into algae colonization and attachment processes with different microorganisms. Our image analyses after nine days of enrichment (Fig. 3) revealed noticeable differences in shapes and sizes ranging from < 1 µm to > 15 µm (Fig. 3Db) of bacteria, possible aggregates, and organic attachments (Fig. 3Ca,Da) and specific characteristics on the algae surface (Fig. 3A). Interestingly, our images show an increased abundance of bacteria occurred in disrupted parts of the algae surface compared to less degraded areas (Fig. 3C).

**Figure 4:**
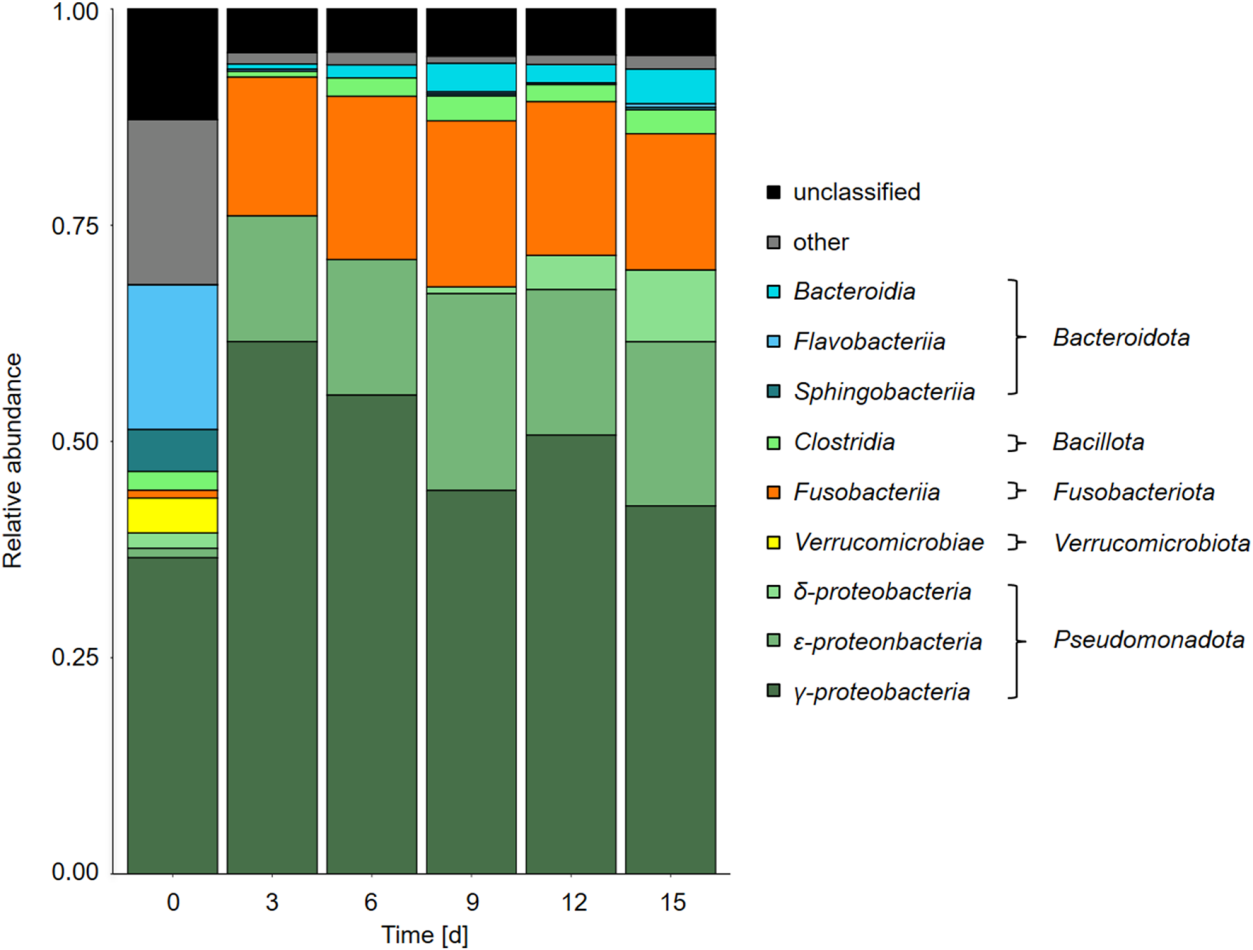
Bacterial community dynamics in *F. vesiculosus* enrichment cultures. 16S rRNA gene amplicon analysis of the microbial community over 15 days on a MiSeq (Illumina) sequencer. Bacterial classes with > 1% of the overall reads are displayed. One-step amplification was utilised to create 16S rRNA gene amplicon libraries. These libraries were subjected to paired-end sequencing using an Illumina MiSeq sequencer (MiSeq Reagent Kit v2, 500 cycles, product number: MS-102-2003), resulting in 2 × 251 bp reads.

We investigated the bacterial community in *F. vesiculosus* enrichment cultures through 16S rRNA gene amplicon analyses. Samples were taken over 15 days (d) (Fig. 4, total combined and filtered reads: 826,738 reads). 16S rRNA gene amplicon results of d0 display the naturally attached bacterial community associated with *F. vesiculosus* on the Baltic Sea shore during November 2022. Above a cut-off of 1% of all bacterial class-associated reads, nine different classes from 5 phyla were identified. In our dataset, *γ-proteobacteria* represented the dominating class with 36.6% of the reads, followed by *Flavobacteriia* with 16.7%. The initial diversity of the bacterial community, measured by Shannon (ShDiv = 4.284) and Simpson (SiDiv = 0.957), decreased as enrichment began. By d3, the diversity further decreased (ShDiv = 2.626, SiDiv = 0.894), resulting in a community with a dominant fraction of *γ-proteobacteria* (61.5%). *Fusobacteriia* abundance increased from initial 0.9% to 16% on d3. Meanwhile, the initially more extensive community of *Flavobacteriia* decreased to 0.2% relative abundance. In the following days of the enrichment, the composition of bacterial classes showed minor fluctuations (d06 ShDiv = 2.924, SiDiv = 0.927; d09 ShDiv = 2.947, SiDiv = 0.922, d12 ShDiv = 2.98, SiDiv = 0.921; d15 ShDiv = 3.13, SiDiv = 0.935).

The change was even more noticeable on the phylum level. Overall, the ratio of *Pseudomonadota* ranged between 58.9% on d0 and 76.0% on d3, making up more than half of the bacterial community over the entire 15-day period. In contrast, members of *Bacteroidota* represented 21.6% of the bacterial composition on d0 but rapidly decreased to 0.8% on d3 while making up 4.7% on d15.

Furthermore, the number of identified genera decreased from 170 on d0 to 26 on d15. During the ongoing enrichment, five genera above 1% relative abundance dominated the community: *γ*-*proteobacteria* species of *Marinomonas*, *Vibrio* and *Psychromonas*, *ε-proteobacteria* species of *Arcobacter* as well as *Fusobacteriota* species of *Propionigenium*. Interestingly, *Psychromonas* were dominant in the initial sampling at d0 with a 21.1% relative abundance ratio. At the beginning of the enrichment process, the ratio of this genus decreased to 1.9% on d3 but ranged between 13.8% and 5.7% in the following 12 days. Besides this, *Marinomonas* species increased in relative abundance from 4.3% on d0 to the highest ratio within the bacterial community of all genera on d3 with 33.5%, followed by fluctuating rates between 15.0 and 20.0%. In contrast, the starting condition of the enrichment culture revealed that the three genera, *Vibrio, Arcobacter,* and *Propionigenium*, constituted only minor fractions, ranging from 0.8% to 1.4%, within the bacterial community. However, after 3 days of enrichment, their relative abundances increased (d3: *Arcobacter* 14.2%, *Propionigentium* 16.0%, *Vibrio* 22.8%) and then stayed at similar proportions.

Additionally, *Bacteroidota* were represented mainly by *Algibacter lectus* on d0. This species comprised 5.0% of all individuals of the naturally attached bacterial microbiome at the time the macroalgae were collected. In enrichment conditions, this species was not found after only 3 days. Overall, from 40 genera belonging to *Bacteroidota* on d0, only 3 were detected between d3 and d15 in the 16S rRNA gene amplicon dataset (*Dysgonomonas*, *Olivibacter*, *Lutimonas*). The strongest increase was found in the species *Dysgonomonas wimpennyi* of the class *Bacteroidia*, which is only found in a ratio of 0.06% on d0 but then increased up to 3.8% during the enrichment.

### *Bacteroidota* as potential key degrader for *Fucus vesiculosus* cell wall

We sequenced four replicate metagenomes of enrichments after 9 days. Assemblies yielded between 283.16 Mbp to 731.97 Mbp with 399,970 to 1,143,468 assembled contigs, the largest of which ranged from 211.39 Kbp to 350.84 Kbp (Supplementary Table 2).

The phylogenetic classification of 69,146,972 covered gene fragments of the combined metagenome datasets (cut-off > 0.0001 ratio of covered gene fragments) reveals the phylogenetic bacterial composition after 9 days with a Shannon-diversity of 3.998 ± 0.292 and a Simpson-diversity of 0.94 ± 0.016 (Supplementary Fig. 2). Similar to the 16S rRNA gene amplicon data, the dominating fraction of genes originated from *Pseudomonadota* (91.6 ± 2.5%) followed by *Bacillota* (2.5 ± 1.1%), *Fusobacteriota* (2.3 ± 0.7%) and *Spirochaetota* (1.7 ± 0.6%). In contrast, *Bacteroidota* comprised around 1.6 ± 0.4% of the bacterial community in the metagenome. Cluster of Orthologous Genes (COG) analyses provided further insights into the gene composition within the dominant phyla (Supplementary Fig. 3). Genes of the category “carbohydrate transport and metabolism (CTM)” included carbohydrate-active enzymes involved in the degrading process of ACW. The general “DNA/RNA metabolism” category turned out as the major gene fraction in all phyla, followed by genes of the category “Translation, ribosomal structure and biogenesis”. Notably, the *Spirochaetota* had the highest proportion of CTM genes at 8.1 ± 1.1%. In contrast, both *Bacteroidota* and *Pseudomonadota* showed similar proportions, at 6.8 ± 0.9% and 6.7 ± 0.4%, respectively.

How different phyla are involved in the complex degradation process of ACWs is not necessarily represented by the abundance and dominance of specific taxa. Metagenomic analyses indicate the putative degradation potential of specialised bacterial communities, thus emphasizing the importance of understanding their role. With the focus on already-known enzyme families within the glycosyl hydrolases, it is possible to reveal the capability and portion of each phylum on the whole degradation process.

**Figure 5:**
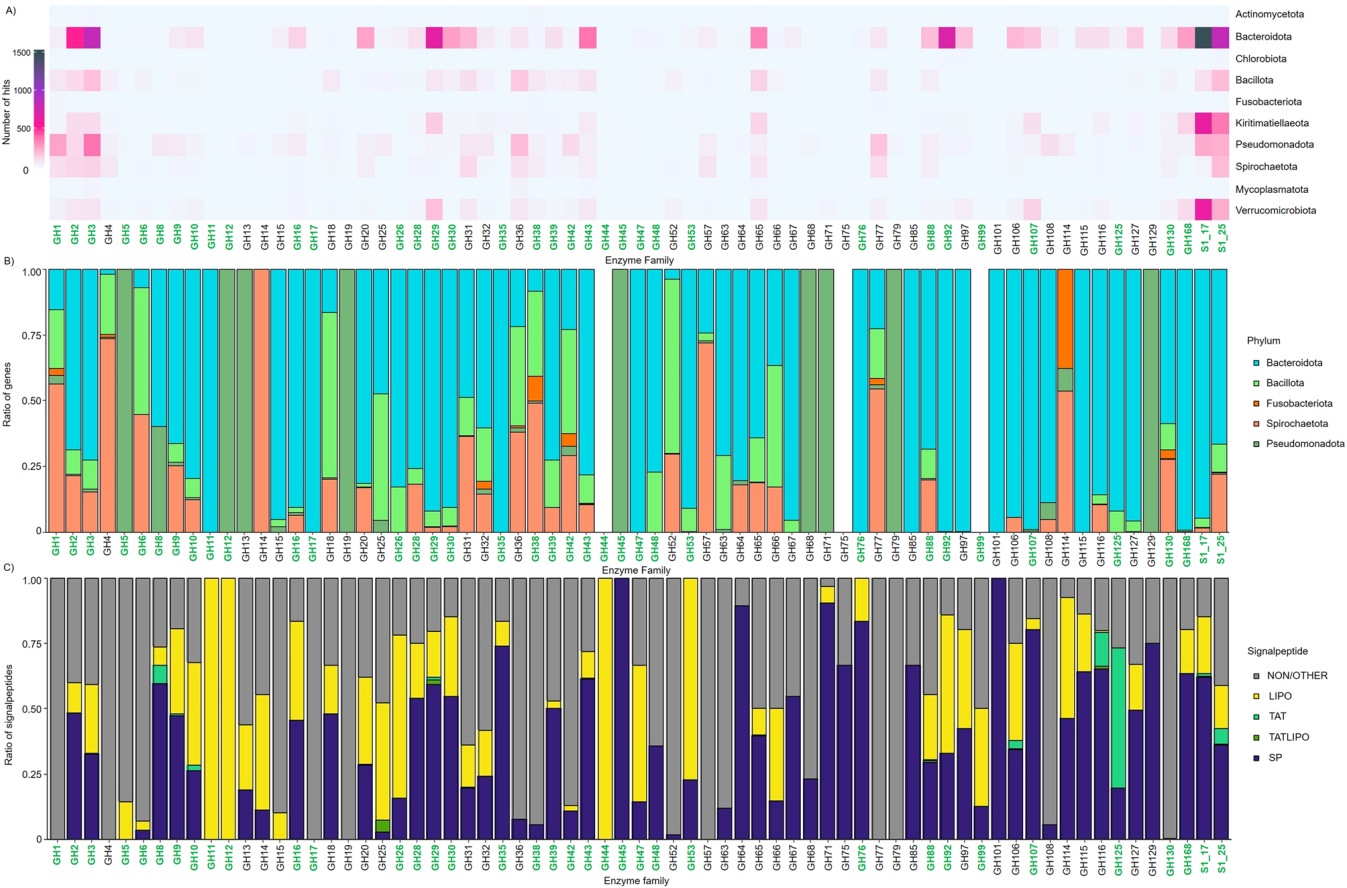
Metagenome analyses of general bacterial gene distributions in GH and sulphatase families based on HMMs. Genes from green highlighted families are involved in algae cell wall polymer degradation. (**A**) Heatmap with number of genes per enzyme family per phylum. (**B**) Ratio of unique genes per enzyme family per phylum normalised by the phylogenetic fraction in the metagenome (Supplementary Fig. 3). Bacterial phyla with a ratio of genes above 1% of the whole metagenome are included. (**C**) Proportions of signal peptides per GH and sulphatase family. Signal peptides were predicted by SignalP v.6.0^2^.

The HMM analysis of coding gene distribution for GHs and SUs families in the metagenome data revealed an elevated number of genes in the *Bacteroidota* compared to all other bacterial phyla (Fig. 5A). A set of 105 different models for GH families led to the identification of 6151 hits with a score above 50 within members of the *Bacteroidota*. All required GH and SU families for the degradation of fucoidan (GH29, GH107, GH168, S1_17, S1_25) were most abundant in this phylum, with SU family S1_17 with the highest quantity of 1467 unique genes. All 8442 unique genes associated with *Bacteroidota* equal 48.8% of the total 17,297 identified GH and SU genes predicted by the HMM analysis of the 10 most abundant phyla.

In total, 1010 GH29 genes (5.84%), 360 GH107 genes (2.08%), 466 GH168 genes (2.69%), 3004 S1_17 genes (17.37%) and 1942 S1_25 genes (11.23%) were predicted. Emphasizing the singular degradation potential of each phylum, normalization was achieved by combining the number of HMM hits per family with the phylogenetic fraction of the corresponding phylum (Fig. 5B). This conversion statistically negates the influence of the phylogenetic abundance and reveals the potential of *Bacteroidota* to degrade ACW even more.

Conversely, *Pseudomonadota*, which is the most abundant phylum based on 16S rRNA gene amplicon analyses and has the highest number of protein-coding genes in the metagenome dataset, exhibited relatively low gene numbers with 2374 hits (13.72%) in the HMM analyses. HMM results assigned most genes to GH1 or GH3 and distributed over 754 species. Both GH families catalyse various substrates compared to specialised families like GH29, GH107, and GH168, which are active exclusively on fucoidan. Interestingly, *Planctomycetota* were represented with a ratio of < 0.0001 of covered fragments in the phylogenetic classification but coded for several putative enzymes active on fucoidan (GH29 195 hits; GH107 5 hits; GH168 114 hits; S1_17 432 hits; S1_25 152 hits). The conducted profile HMM searches showed 1281 hits for *Planctomycetota* in 96 species. *Verrucomicrobiota* exhibited apparent specialisation in the degradation of fucoidan, distributing all 1871 HMM hits (10.82%) across 92 different species, with only 4 species having more than 100 hits*.* For comparison, the 8442 hits of *Bacteroidota* originated from 720 different species, while 17 species of those coded for > 100 unique genes of the conducted HMM.

The species *Lentimonas* sp. CC10 (NCBI:txid2676095) harbours the majority of *Verrucomicrobiota*-associated genes, totalling 394 hits (GH29 46 hits; GH107 52 hits; GH168 31 hits; S1_17 158 hits; S1_25 53 hits). *Lentimonas* sp. CC21 (NCBI:txid2676098) contains 270 hits (GH29 54 hits; GH107 16 hits; GH168 26 hits; S1_17 106 hits; S1_25 23 hits). The *Bacteroidota* species with the most HMM hits was *Mariniflexile fucanivorans* with 404 hits (GH29 49 hits; GH107 5 hits; GH168 26 hits; S1_17 86 hits; S1_25 27 hits) followed by *Formosa algae* with 266 hits. *Reinekea marinisedimentorum* had the highest number of HMM hits among *Pseudomonadota*, totalling 64 putative GH/SU tested genes. Notably, there were no hits for GH29, which is crucial for fucoidan degradation. Likewise, no hits were identified for the 2 SU families. Furthermore, the *Planctomycetota* species *Novipirellula aureliae* codes for 337 putative proteins classified as one of the conducted HMMs (GH29 43 hits; S1_17 86 hits; S1_25 30 hits).

### Implementation of novel GH29 fucosidases

By in silico screening, we predicted 1010 unique amino sequences from the combined metagenomes as putative enzymes of the family GH29 (Fig. 5A). This crucial enzyme family in fucoidan degradation includes α-L-fucosidases (EC 3.2.1.51), α-1,3/1,4-L-fucosidases (EC 3.2.1.111), α-1,2-L-fucosidases (EC 3.2.1.63), α-1,6-L-fucosidases (EC 3.2.1.127) and α-L-glucosidases (EC 3.2.1.-) with 106 characterised enzymes in CAZy in January 2024.

We attempted cloning of 20 GH29 genes, for which a HMM score > 320 was calculated (max. 358.2). The putative genes coding for FUJM18 and FUJM20 were successfully cloned in the plasmid pet21a( +) plasmid and overexpressed in *E. coli* BL21 (DE3). The protein blast revealed a similarity to previously predicted but not characterised GH29 fucosidases of 90.6% to *Prolixibacteraceae bacterium* Z1-6 for FUJM18 and 77.3% for *Arenibacter* sp. F26102. The gene sequences of the cloned vector resulted in a HMM score of 321.4 for FUJM18 and 324.4 for FUJM20 (IMG accession origins Ga0502370_024398_80_1447 and Ga0502371_0001890_2219_3553, respectively). The FUJM18 gene coded for a 444 aa protein, and the FUJM20 gene coded for a 426 aa protein. Both amino sequences were identified to include signal peptides. Excluding those signal peptides, the calculated molecular weight of FUJM18 was 51,534.97 kDA, while the molecular weight of FUJM20 was 47,868.3 kDA. The nucleotide homology search for the FUJM18 scaffold (Ga0502370_024398) results in a similarity of 85.71% for *Prolixibacteracea*. For the FUJM20 scaffold (Ga0502371_0001890) we could analyse a significant BLAST homology for *Flavobacteriaceae*.

### Structure prediction and activity assay

**Figure 6:**
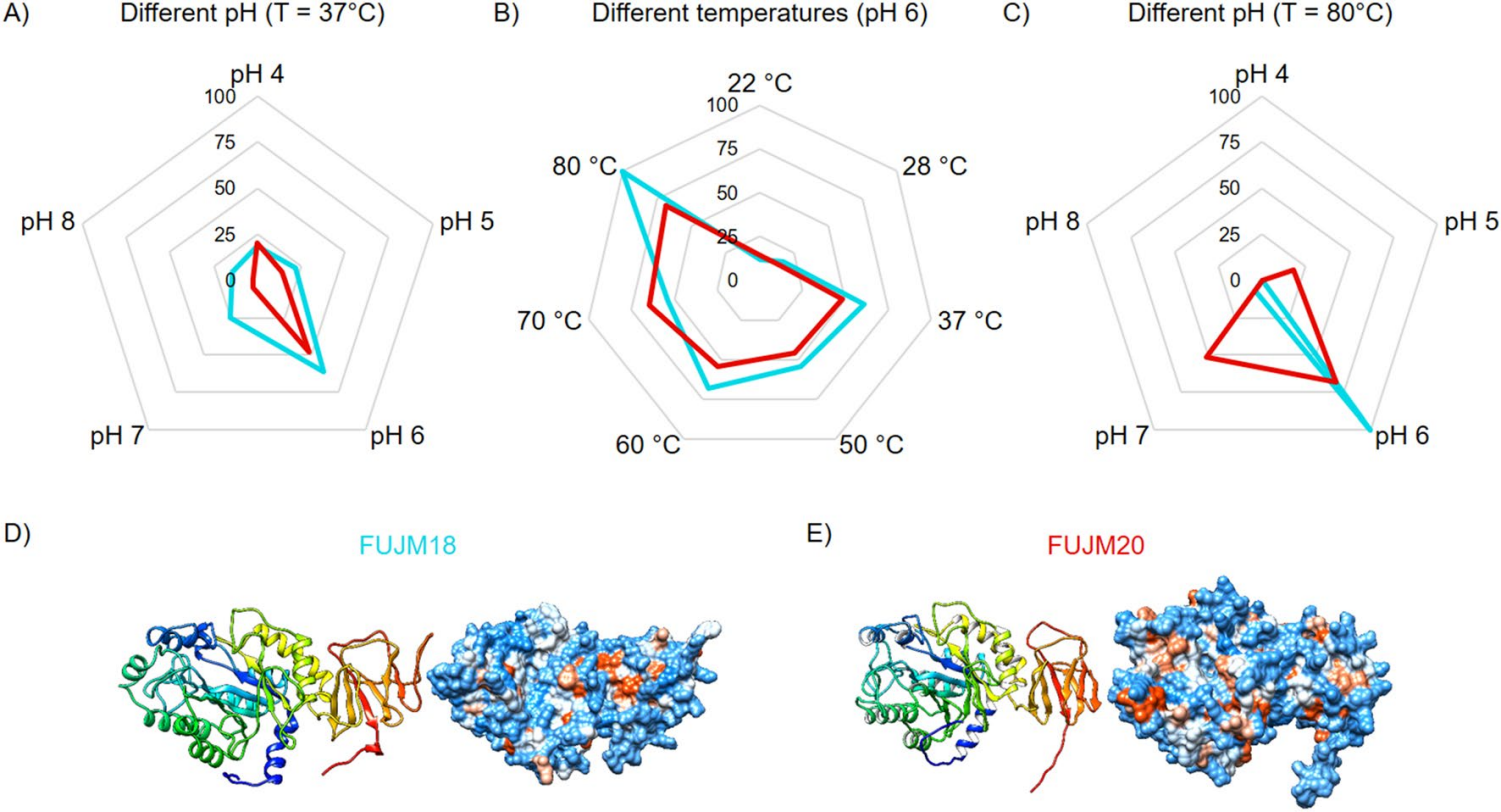
Characterisation of FUJM18 and FUJM20. Relative hydrolysation activity measured with 4-Nitrophenyl-α-L-fucopyronase of the GH29 enzymes FUJM18 and FUJM20 in triplicates. 100% relative activity equals 61.18 U*mg^−1^ enzyme activity (Supplementary Table 3). (**A**) Determining the optimal pH value for the enzyme activity at 37 °C. FUJM18 peaks at pH 6 with 61.12 ± 27.29% relative activity (37.39 ± 16.69 U*mg^−1^). (**B**) Determining the optimal temperature for the enzyme activity at pH 6. FUJM18 peaks at 80 °C with 100 ± 20.9% relative activity (61.18 ± 12.79 U*mg^−1^). (**C**) Determining the optimal pH value for the enzyme activity at the optimal temperature of 80 °C. (**D**) Predicted structure of FUJM18 by Alphafold 2. E) Predicted structure of FUJM20 by Alphafold 2^3^.

**Figure 7:**
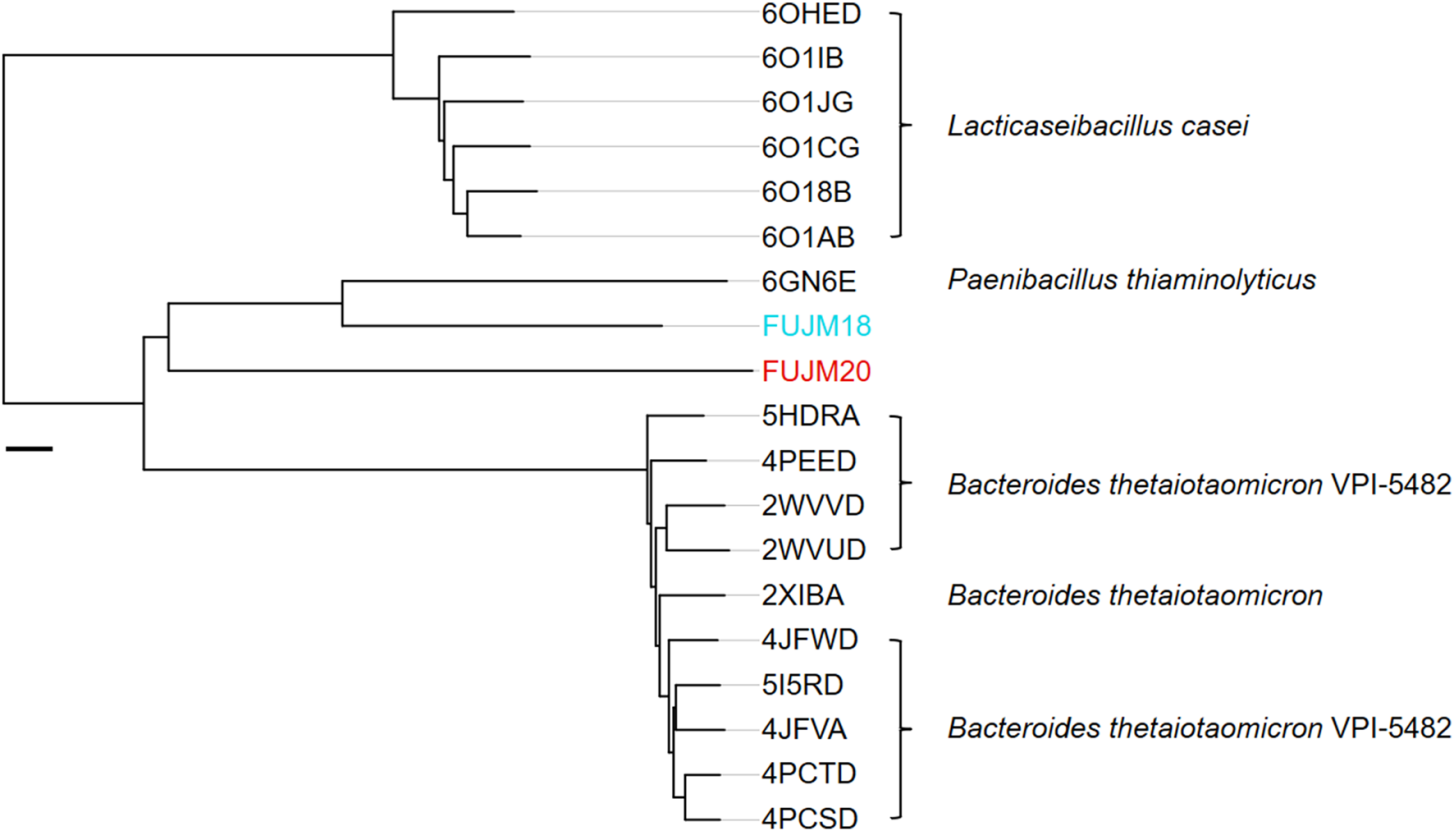
Phylogenetic tree of FUJM18 and FUJM20 based on protein structures in an all against all analyses of the Dali Server and PDB database. Chosen structures have a similarity cut-off of an RMSD value < 2.2. Similarity distance is shown in Z-score; the scale bar indicates a distance of 2.

In activity assays with *p*NP-α-L-Fucopyranose (*p*NP-α-L-Fuc), FUJM18 showed higher activity on the substrate compared to FUJM20. In contrast, FUJM20 showed a more comprehensive range of activity at different pH-values as well as temperatures. FUJM18 and FUJM20 are thermostable α-L-fucosidases with an optimal temperature of 80 °C at pH 6 in 0.1 M sodium citrate buffer (Fig. 6A–C, Supplementary Table 3). FUJM18 was active with a maximum of 61.18 ± 12.79 U*mg^−1^, FUJM20 with 41.77 ± 1.34 U*mg^−1^. The structure prediction of both proteins displayed a beta-sheet comprised of six interleaved beta strands on the C-terminus (Fig. 6D,E). The protein database (PDB) structure comparison revealed that both enzyme structure predictions are most similar to the entry α-L-fucosidase isoenzyme 1 from *Paenibacillus thiaminolyticus* chain D (6GN6), with the highest structural similarity of FU18JM at a Z-score of 51.6 (Fig. 7, Supplementary movie 1, Supplementary movie 2)^48^. FUJM18 and FUJM20 have a Z-score of 42.9 (Supplementary movie 3). Previous research of crystal structures of the structural most similar enzymes was found to form multimers like the hexamer of α-L-fucosidase isoenzyme 1 from *Paenibacillus thiaminolyticus*.

In summary, the result of our enrichment culture degradation approach shows the outstanding potential of bacterial communities in ACW carbohydrate hydrolysation and the robust pipeline of the “omics”-based gene screening to identify genes/enzymes and species to implement sustainable resources in industrial processes. We identified over 13,000 putative glycosyl hydrolases from 34 enzyme families involved in the degradation process of at least one ACW carbohydrate and over 4900 putative sulphatases of 2 enzyme families. The genomes of *Bacteroidota* species contained an outstanding majority of the identified protein sequences. However, in the microbial community, the fraction of *Bacteroidota* individuals is suppressed by the abundance of *Pseudomonadota*.

As HMM revealed the gene candidates with the highest potential for fucoidan degradation, we could clone, test, and characterise two active, thermostable α-L-fucosidases. These enzyme structures are most similar to an α-L-fucosidase synthesised by a *Bacillota* species. However, protein–protein blast results revealed the highest sequence coverage for *Bacteroidota* species.

## Discussion

The intricate, non-consistent structure of ACWs requires an equally complex enzymatic degradation machinery. Considering all ACW carbohydrates, we showed the presence of certain bacteria encoding for certain enzymes from at least five different classes associated with the degradation process: 49 Glycosyl hydrolase families (GH), 24 Polysaccharide lyase families (PL), 4 sulphatase families, 31 Carbohydrate binding module families (CBM) and 7 carbohydrate esterase families (CE) (Supplementary Table 4)^34,49^.

With diverse structure variations, the backbone of fucoidan is mainly made up of α-1,3/1,4-L-fucose. The carbohydrate structure is divided into homofucan and heterofucan. While homofucan is one chain of α-1,3 or alternating α-1,3/α-1,4-linked L-fucose with sulphuric acid groups at C2, C3, or C4, the structure of heterofucan is more complex with additional monosaccharides like galactose, xylose, mannose, glucose, rhamnose, and uronic acid that enable crosslinked molecule structures (Supplementary Fig. 4A)^49,50^. However, laminarin has a less complex structure of β-1,3-D-glucose chains with occasionally β-1,6-D-glucose crosslinks^51^. Besides *Phaeophycean*, to which *Fucus* belongs, laminarin is also a cell compound in *Bacillariophyta*. Another brown alga-associated polysaccharide is cellulose, also found in *Chlorophyta* and as the primary cell wall component in land plants. It consists of non-crosslinked chains of β-1,4-D-glucose^52^. Alginate is the fourth polysaccharide, with brown algae species as the only producers within the kingdom plants. Its complex structure consists of a copolymer with varying configurations of 1,4-linked β-D-mannuronate and α-guluronate residues^53^.

Further, ACW carbohydrates are carrageenan, agar, and porphyran in red algae; ulvan in green algae; mannan and xylan in red and green algae; and pectin in green algae and *diatoms*^54^. Considering our understanding of crucial enzyme classes and families (Supplementary Table 4) and positive results from HMM analyses, particularly in GH families associated with laminarin and cellulose degradation (Fig. 5A), it is reasonable to infer that the enriched bacterial community observed is proficient in degrading various carbohydrates. However, the higher number of hits per gene coding for enzyme families active on fucoidan exceeded those for the other mentioned substrates, presumably due to the dominating component of fucoidan in the cell wall carbohydrate composition of *F. vesiculosus*.

The metabolic catalysis of fucoidan is well studied and consists of a cascade of GHs, CBMs and sulphatases (Fig. 1)^21,38–40^. The sulphated oligosaccharide homofucan is hydrolysed by endo-α-1,3/1,4-fucoidanase from GH families GH107 and GH168, followed by exo-fucose-2/3-sulphate or (fucan)-2/3-O-sulfohydrolase of families S1_17 and S1_25. Additionally, reported active sulphatases are found in families S1_15 and S1_16^21^. Further, α-L-fucosidases classified as GH29, GH95, GH141 and GH151 degrade intermediate oligosaccharides down to the desulphated monosaccharide L-fucose, which is then finally metabolised in the TCA cycle^49^.

During the degradation process of ACWs, we could measure the increase of released and reduced sugars in the supernatant of the enrichment cultures with the *p*HAH assay over about 7–9 days, followed by a decline to around half of the concentration of the maximum values (Fig. 2B). In a multi-step degradation process, this time point marks a shift in the balance between bacteria breaking down L-fucose and other monomers and the bacteria supplying these monomers along with the exposed carbohydrates. Consequently, the increasing release of ACW compounds led to a deconstruction of the algae in the enrichment culture (Fig. 2A). Regarding industrial usage of algae as an alternative resource, this biomass degradation is only one step of a complex process. Future research needs to address multiple challenges in algae farming and cultivation upscaling. Harmful threats for valuable, farmed algae, like complex zooplanktonic grazer communities that feed on the algae and induce morphologic reactions, are critical for the industrial success of this resource^55–57^. The degradation process will not occur to the extent of a necessary state, as long cultivation is not productive, and energy and commercial efficiency are lacking. This study does not target or control different zooplankton grazers, but it is anticipated that they will influence both the degradation process and the overall community composition in the mesocosms.

Focusing on bacterial metabolism, energy uptake and conversion are reflected in species abundance. The increasing abundance of *Pseudomonadota* revealed by 16S rRNA gene amplicon analyses hints that this phylum is the most efficient in metabolising at least the sugar monomers into their TCA cycle as well as *Fusobacteriota* and *Bacillota* (Fig. 4). Other influences like temperature, oxygen conditions, medium volume of the enrichment cultures, and the bottle effect are critical for the bacterial community dynamics as well. Initial testing of different temperatures and shaking intensities helped design reproducible degrading conditions, but a bottle effect that favours fast-growing *γ-proteobacteria* is expected^58^. Especially the initial dominating fraction of *γ-proteobacteria* shows their association to algae carbohydrate substrates. Furthermore, recent studies of *Psychromonas* underline this association as incubation with pectin and alginate favoured their relative abundance^59^. In contrast, this genus declined in our study’s experimental incubation and substrate conditions. However, the impact of *γ-proteobacteria* in the enzymatic composition of the enrichment culture might not be redefected as this study solely focuses on the listed GH and SU families. An extended analyses of the metagenome would presumably reline the role of this bacterial class and explain their abundance and their influence to the bottle effect in enrichment cultures. Further experiments of the investigated microbiome in enrichment cultures without the algae as substrate were not carried out, but could help to determine the influence of the bottle effect.

An increasing abundance of *δ-proteobacteria* and *ε-proteobacteria* indicates the micro-anaerobic conditions and is a disadvantage for aerobic *Bacteroidota*. Furthermore, the putrid smell emerging from the enrichment cultures hints at reducing *ε-proteobacteria*. *δ-proteobacteria* and *ε-proteobacteria* were previously found in anaerobic environments in the Baltic Sea^60,61^*.* The relatively slow shaking and the lightly sealed flask may have caused the low oxygen concentration. Additionally, a biofilm formation on the culture surface could have weakened the mixing effect by altering the surface tension. The activity in the enrichment cultures usually ends after 25 to 30 days (Fig. 2A). As we found a loss of approximately one-fifth of the initial algae wet weight, the microbial degradation is not efficient enough to degrade the major part of the algae. The side effects described above inhibit the degrading community until a turnover event stops the enrichment. Besides anaerobic conditions, these effects could depend on phages that kill the bacteria culture, the release and rising concentration of toxic compounds through cell lysis, inter- and intraspecific competition, nutrient limitations, and general unfavourable medium conditions. Further research must be done to target these challenges to make algae farming possible and lucrative at a high level. With our findings, we contribute to the general understanding of the enrichment culture dynamics and interactions.

*Bacteroidota* species are prominently known for their degrading capabilities in natural systems with a high potential in several biological hydrolysing applications^62,63^. The screening approach for metagenomes employed in this study has identified novel putative GH enzymes and other proteins associated with ACW hydrolysis, thereby reinforcing the robustness of these findings. The metagenome approach enables the accessibility of genes of nonculturable bacteria. By using HMM analysis, we were able to underline the exceptional potential of the *Bacteroidota* to degrade sulphated ACW carbohydrates compared to all other phyla. Previous research already described the expression of α-L-fucosidases of GH29 in *Bacteroidota*^64^.

Furthermore, our results support the previously landmark study of Sichert et al., which assigns species belonging to the genus *Lentimonas* from the phylum *Verrucomicrobiota* to express hundreds of genes involved in the fucoidan degradation^21^. In the metagenome of the enrichment culture after 9 days, we can further confirm and strengthen this finding. We found 782 unique genes with HMM analyses originating from *Lentimonas* spp. (mainly *Lentimonas* sp. CC10 NCBI:txid2676095 and *Lentimonas *sp. CC21 NCBI:txid2676098) with putative enzyme functions of the analysed 67 GH families and 2 SU families. Interestingly, the HMMs assign 289 genes of those to the enzyme families GH29, GH107 and GH168 and 386 genes to the enzyme families S1_17 and S1_25. With these results, we can amplify the previously described degrading capabilities of *Verrucomicrobiota*, especially *Lentimonas* spp.^21^. However, putative fucoidan-degrading genes were associated with *Bacteroidota*. Furthermore, the HMM top scores of the genes from the ACW degrading families originate from this phylum. This leads to an increased variability and possible adaptation to the different conditions and structures of algae cell wall carbohydrates of a *Bacteroidota* community in natural systems. The number of hits throughout multiple GH and SU enzyme families enables the degradation capability of a purified substrate like fucoidan and a composition of multiple natural carbohydrates. Certain specialised *Bacteroidota* species provide multiple potential fucoidan-active enzymes. These species represent important candidates for future approaches to single and mixed-species degradation. So far, previous research investigated the degradation processes of the described *M. fucanivorans*^65^.

**Figure 8:**
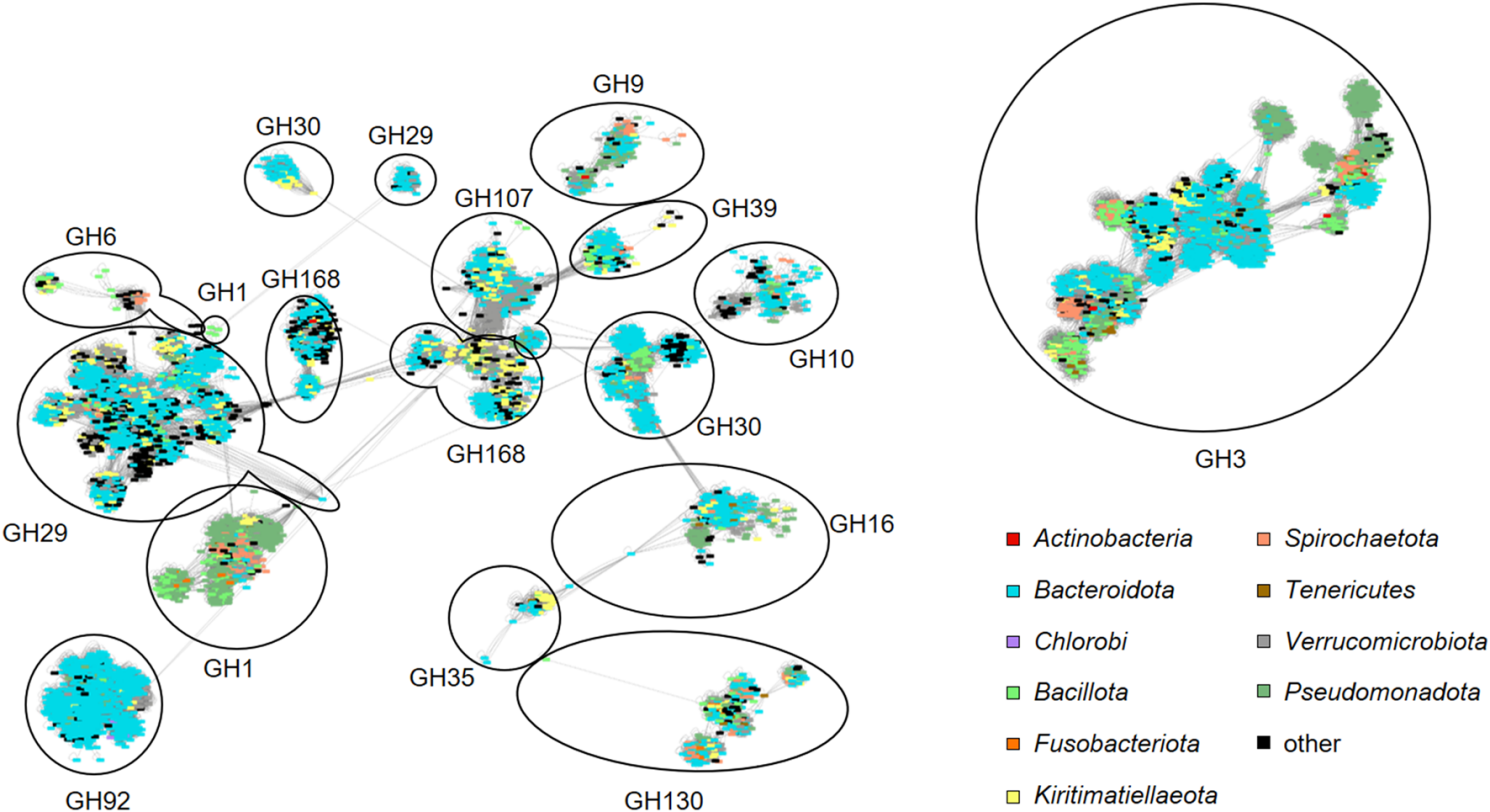
Sequence similarity network of Hidden Markov Model search for glycosyl hydrolases in metagenomic datasets of *Fucus vesiculosus* enrichment culture microbiomes. Filtered GH families are involved in the degradation of *F. vesiculosus* cell wall carbohydrates. Coloured genes indicate the phylum. Data analysed and displayed with Cytoscape v.3.9.1^4^.

These results lead to an imbalance between the abundance of *Pseudomonadota* and *Bacteroidota* and their carbohydrate-degrading activities in *F. vesiculosus* enrichment cultures, especially noticeable by normalising the number of HMM hits (Fig. 5B). We assume this relationship depends on these phyla’s L-fucose and general sugar monomer metabolism, which is more efficient in *Pseudomonadota*. The already discussed enrichment culture effects for fast-growing *γ-proteobacteria* also influence this development^58^*.* The *Bacteroidota* species find a rich source of degradable carbon substrates within the ACW carbohydrates and use energy as a trade-off to synthesise the mandatory enzymes, most of which are then secreted from the cell to the algae cell surface (Fig. 5C). This degradation leads to an increase of different reduced sugar chains on the enrichment culture medium. However, the payoff is for *Pseudomonadota* as they invest less energy on polysaccharide degradation but benefit from an increased energy intake compared to *Bacteroidota*. Influences of selfish uptake also apply to this interaction as this behaviour is found over the whole water column in the bacterioplankton for the *Bacteroidota*, *Pseudomonadota*, and *Verrucomicrobia* beyond others^66^. While our sequence-based analysis shows a cluster of the different GH families and further reveals the dominance of *Bacteroidota* in general, genes of family GH1 are mainly in *Pseudomonadota* genomes (Fig. 8). GH1 itself contains a variety of 27 enzyme activities for a high number of different substrates. It is not as specialised as the GH29 family, which only comprises five enzymatic activities, including four different types of α-L-fucosidases.

In summary, we hypothesise that the key and base findings of this study will provide knowledge to an efficient degradation for algae cell wall carbohydrates. So far, we got a degradation rate of 21.52 ± 0.14% in wet weight loss of the plant. For similar future degradation approaches, monitoring and adjusting the sugar concentration in the media may increase the degradation efficiency by creating a beneficial environment for *Bacteroidota* while *Pseudomonadota* is inhibited. However, if these adjustments overcome or suppress the discussed enrichment culture, challenges have to be addressed in future studies.

GH29 successfully degrades ACW by catalytically hydrolysing L-fucose dimers and detaching L-fucose monomers from sulphated polymers during the degradation process (Supplementary Fig. 4B). The present approach of metagenome mining by HMMs in combination with enrichment cultures is an efficient way of finding novel, valuable enzymes. In our case, FUJM18 and FUJM20 represent thermostable and functional enzymes that the HMM identified for GH29. As described, the fucoidan is supposed to be pre-treated by enzymes of GH107 and GH168 as well as S1_17 and S1_25. With the *p*NP-α-L-Fuc assay, it was possible to confirm the enzymes as α-L-fucosidases.

Structural similar entries for α-L-fucosidase isoenzyme 1 from *Paenibacillus thiaminolyticus* (6GN6) and α-L-fucosidase complex from *Lacticaseibacillus casei* (6O1A) in the RCSB PDB Protein data Bank (RCSB.org) suggest that the proteins could form multimers with an active site in the centre of the resulting complex (Fig. 7, Supplementary movie 1, Supplementary movie 2)^48,67,68^.

Overall, we introduced two powerful enzymes with high industrial potential as thermostable candidates for processing marine organic matter.

## Conclusion

In summary, our study provides detailed insight into the microbial degrading dynamics in macroalgae enrichment cultures and offers solutions for future high ACW degrading approaches. Our omics analysis shows thousands of putative GH and SU enzymes that could be involved in degrading various carbohydrates and are specialised to fucoidan when attached to *F. vesiculosus* by *Bacteroidota*. As in natural marine surface conditions, we detected a microbial community dominated by *Pseudomonadota* in artificial microcosm experiments but with a declining abundance of *Bacteroidota*^69^. *Pseudomonadota* is likely to be significantly more efficient in metabolising the resulting sugar mono- and dimers, even though their contribution to the initial degradation process is likely minor. The observation of *Bacteroidota* as the potential main driver of fucoidan degradation and *Pseudomonadota* as the dominant phylum sets this assumption. Additionally, HMM scores reveal that the top-scoring genes of enzyme families associated with the fucoidan degradation also originate from *Bacteroidota*.

Future research experiments should establish a universal mixture of ACW degrading proteins found by sequence-based screening of omics-based approaches. This enzyme mixture will enable algae to be a novel resource for industrial, sustainable use. Exemplary, were able to successfully identify and introduce 2 novel, thermostable α-L-fucosidases of GH29 using our conducted screening method.

## Methods

### Sampling and enrichment

Fresh environmental samples of *F. vesiculosus* were collected at the shore of the Baltic Sea within the tidal zone of the Kiel fjord (54°21′56’’ N, 10°11′43’’ E) and transferred to the lab in humid conditions. The samples were stored at 4 °C before processing.

The algae material was rinsed with VE Water, and attached animals like barnacles were removed or excluded, as well as other plant material. For designing the final degradation cultures, initial testing with different enrichment conditions was performed and observed for degradation and microbial activity. These tests included altering temperatures, shaking speeds, oxygen conditions and volumes of medium and algae. Further, artificial inoculations with different soil samples and animal feces (horse, elephant) were tested. For the artificial inoculations, *F. vesiculosus* was autoclaved in advance. In all tested conditions *F. vesiculosus* served as carbon source.

In the final approach applied in this study, 50 g of the algae were transferred into 500 ml Schott flasks containing 300 ml of synthetic seawater (Tropic Marin Classic Meersalz, Hünenberg, Switzerland) with a salinity of 15, mimicking the conditions found in the upper water layer of the Kiel fjord. No additional carbon source was added besides the algae and the minor amounts of sea salts.

The Schott-flasks were lightly sealed to keep micro-anaerobic conditions. The enrichment cultures were kept at 22 °C and slowly shaken on a GFL™ 3015 laboratory shaker at 60 rpm. The enrichment cultures were observed over time up to 30 days. Before aliquot samples were taken for further analyses, the enrichment cultures were manually shaken by hand for a homologous distribution of the components in the supernatant. Fluid samples were transferred and stored at − 70 °C until further procedures. After every conduction, the removed volume of supernatant of the enrichment cultures was refilled with fresh medium.

### Microbial community analysis

For genomic studies, 1 ml of the supernatant was centrifuged for 5 min at 12,500 g, followed by an isolation of the DNA with the Zymo Research Quick-DNA Fungal/Bacterial Kit (Zymo Research Europe GmbH, Freiburg Germany). 16S rRNA gene amplicon approaches were conducted to investigate the bacterial community dynamics in the enrichment cultures over time using barcode primers 515f. and 806rcbc for 16S rRNA amplicon analyses (1 × PCR ingredients: 2 µl Buffer B(10 ×) (ThermoFisherscientific, Waltham, MA; USA); 0.4 µl 10 mM dNTPs; 2 µl 25 mM MgCl_2_, 0.5 µl,10 µM FW/RV-Primer (Supplementary Table 5), 0.6 µl DMSO, 0.2 µl DCS-Taq polymerase (Qiagen, Venlo, Netherlands), 15 µl (2.5 ng*µl^−1^) template DNA, 3.8 µl H_2_O)^70^. 16S rRNA gene amplicon libraries were generated by applying a one-step amplification protocol (PCR conditions: 95 °C 5 min, 25 cycles of 95 °C for 45 s, 55 °C for 30 s, 72 °C for 45 s, final extension 72 °C for 2 min). Libraries were paired-end sequenced on a MiSeq (Illumina) sequencer (MiSeq Reagent Kit v2, 500 cycles, product number: MS-102-2003) with 2 × 251 bp (plus 1 × 12 bp Index read)^71^. After sequencing, the bioinformatics pipeline performs sequence demultiplexing, adaptor and primer trimming. The 50-300 K sequencing reads per sample were analysed using the 16S Metagenomics Software version 2.6.2.3 (Illumina). This allow us to analyse OTUs (Operational Taxonomic Units) from different time points, comparing taxonomies via a curated database of 16S rRNA gene entries (greengenes.secondgenome.com, based on OTUs and QIIME) (Supplementary Table 5)^72–76^.

Scanning electron microscopy (SEM) images of *F. vesiculosus* samples after 9 days in enrichment cultures were provided for insights into degrading processes and microbial communities. It was performed as previously published^77,78^. The samples were fixed in paraformaldehyde (1%) and glutaraldehyde (0.25%) (30,525-89-4 and G4004, Merck, Taufkirchen Deutschland), dehydrated by ascending alcohol series and dried at the critical point with Balzers CPD 030 Critical Point Dryer (BAL-TEC, Schalksmühle, Germany). After coating samples with gold/carbon using a sputter coater SCD 050 (BAL-TEC, Schalksmühle, Germany), scanning electron micrographs were taken with a LEO 1525 (Zeiss, Oberkochen, Germany).

### Metabolic dynamics in enrichment cultures

Monitoring the general algae degradation fractionating of the organic matter, bacterial growth, and biological activity, as well as was the release of organic compounds was archived by measuring the optical density at 600 nm (OD_600nm_) in triplicates. An increase or change of turbidity is assumed to correlate with released degradation products and microscale fractions of the algae but also with bacterial growth and can estimate microbial degrading activities. Also, the wet weight loss after 20 days in the enrichment was measured in triplicates. For this, the whole enrichment culture was filtered through a 1 × 1 mm mesh as every minor plant fraction was considered a degradation product.

The expected hydrolysation of ACW polysaccharides leads to a change in the concentration of sugar monomers and reducing sugar ends. The *para*-hydroxybenzoic acid hydrazide (*p*hah) assay enables the quantification of reducing sugar equivalents and detects open forms as well as hemi-acetal and hemi-ketal of carbohydrates. Therefore, two reagents (A: 0.3 M 4-hydroxybenzhydrazide, 0.6 M HCl; B: 48 mM trisodium citrate, 10 mM CaCl_2_, 0.5 M NaOH) were mixed 9:1 to a final volume of 1 ml. The mixture was then supplemented with 50 µl of enrichment culture supernatant and heated at 100 °C with constant mixing. Additionally, L-(-)-Fucose (Sigma-Aldrich, Saint Louis, MO; USA) were used as an equivalent standard and diluted in the synthetic seawater (Tropic Marin Classic Meersalz, Hünenberg, Switzerland) with a salinity of 15. The absorbance determined the concentration at 410 nm. The values were fitted to the L-fucose standard curve prepared in triplicates. This method was conducted in triplicates to the corresponding enrichment culture of the OD measurement.

UHPLC-ESI-HR-MS2 data for metabolomic analysis was recorded using an Acquity I-class UHPLC (Waters, Milford, MA, USA) coupled to a PDA detector and a Vion IMS QToF (Waters). The chromatographic separation was performed using an Acquity C-18 UPLC column (1.7 μm, 2.1 mm × 100 mm; Waters). Mobile phases consisted of acetonitrile (HiPerSolv, VWR) for mobile phase A and pH_2_O produced by the in-house Milli-Q system as mobile phase B, both containing 0.1% (v/v) formic acid (33,015, Sigma). The gradient was run from 10 to 100% B over 13 min at a flow rate of 0.45 ml*min^−1^. The MS was run in DDA mode, recording a *m/z* range from 150 to 1000 using a desolvation temperature of 350 °C, a source temperature of 120 °C and a cone voltage of 0.8 kV. Samples were run in ESI + and ESI- ionisation mode. The collision energy for the generation of product ions in MS2 was set to a mass-dependent ramp from 20 to 60 keV, and N_2_ was used as collision gas. The data was processed and analysed using UNIFI 1.9.4 (Waters). Metabolomic analyses, peak picking and principal component analysis were done using Progenesis QI (Waters). Freeze-dried samples were dissolved in 80% (v/v) Methanol *aq*. (HiPerSolv, VWR) and 5 µL of the sample were injected for analysis.

### Metagenome sequencing of *F. vesiculosus* enrichment cultures

Metagenomes were prepared of four biological replicates of the enrichment cultures. Interim results of the metabolic data (*p*hah, OD_600nm_, visual observations) suggested a timepoint of 9 days after the start of the enrichment for the highest degradation activity. This determined the day of sampling the metagenomes. The DNA of 1 ml supernatant from the enrichment cultures was isolated with the Zymo Research Quick-DNA Fungal/Bacterial Kit (Zymo Research Europe GmbH, Freiburg Germany). Library preparation and sequencing were performed at the Leibniz Institute of Virology (LIV, Hamburg). For library preparation, 1 ng total DNA extracted from enrichment cultures at d9 was used as input material by applying the Nextera XT DNA Library Preparation Kit (Illumina, product number: FC-131–1096) according to manufacturer’s instructions. Libraries were paired-end sequenced on a NextSeq 500 (Illumina, San Diego, CA; USA) sequencer (NextSeq 500/550 High Output Kit v2.5, 300 Cycles, product number: 20024908) with 2 × 151 bp (plus 2 × 8 bp Index reads). Demultiplexing with bcl2fastq (default settings) yielded approximately 112–143 mio reads per sample.

Sequence reads were processed with fastp (v0.21.0) to remove sequences originating from sequencing adapters and sequences of low quality (Phred quality score below 20) from the 3' end of the sequence reads^79^. Processed reads shorter than 40 bp were discarded. The remaining reads were assembled using SPAdes (v3.15.3) in metagenomic mode^80^.

### Metagenomic dataset analyses and processing

Profile Hidden Markov Model searches were performed as described previously^81^. Shortly, we extracted the curated HMMs related to glycosyl hydrolases (GH*.hmm, Glycohydro*.hmm, Glyco_hydro*.hmm, and Glyc_hyd*.hmm; Supplementary Table 6) from the InterPro/Pfam database in 03/2022^82^ as well as sulphatases S1_17 and S1_25 from the Sulfatlas database in 03/2023^35,36^ and searched all CDS from each metagenome with hmmsearch from the HMMER v.3.3 software package^83^. Additionally, two HMMs (GH107 and GH168) were designed with the enzymes extracted from the CAZy database^34^. If a protein-coding region in the metagenome was associated with several enzyme families, the highest HMM score was taken for the final assignment. The reporting cut-off was set at an e-value < E-10. N-terminal secretion signal prediction was performed with SignalP v.6.0^84^. The phylogenetic affiliation of each potential hit was inferred from the best BLASTp hit in the NCBI nr-database^85^ using DIAMOND v.2.0.15^86^. “All against all” alignments were performed with DIAMOND BLASTp, reporting only alignments with e-value < 0.01. In the displayed data of number of genes, the four metagenomic datasets were combined, including only unique gene sequences. Visualisation of the Sequence Similarity Network (SSN) was conducted in Cytoscape v.3.9.1^87^ applying the “Prefuse Force Directed Layout (none)” as described elsewhere^88^. Taxonomic classification was carried out with Kraken2 (v2.12) (KRAKEN) in combination with Bracken (v2.6.2) (BRACKEN)^89,90^. R v.4.2.3 was conducted for plotting and the visualisation of metabolic, phylogenetic and metagenomic results mainly using the packages ggplot2 v.3.4.4 and ComplexHeatmap v.2.16.0^91–93^. Additionally, R v.4.2.3 served to perform reported cut-off applications (Kraken analyses 0.0001 ratio of covered fragments). The vegan package v.2.6—4 was used for diversity calculations^94^.

### Gene cloning and protein expression

Putative substrate degrading genes were cloned in the pet21α( +)-plasmid and transferred into *Escherichia coli* BL21. The metagenomic DNA was extracted with the Zymo Research Quick-DNA Fungal/Bacterial Kit (Zymo Research Europe GmbH, Freiburg, Germany). Primers were designed for the top-scoring genes of the HMM for enzyme family GH29 involved in the fucoidan degradation cascade and extended with restriction enzyme sites for ndeI and xhoI and a removed stop codon, which leads to the addition of the His-tag to the insert in the pet21α( +) plasmid. Potential signal peptides were predicted with SignalP v. 6.0 and excluded from the final insert, as both successfully cloned enzymes were found to include a signal peptide (Sec/SP1)^84^. The resulting primer sequences for the genes coding for the protein FUJM18 are 5’-CAACAAAAAATATGGAACGAAACCG-3’ (forward) and 5’-TTTCAGAAATAGCTCAATCACGG-3’ (reverse) and for FUJM20 5’-CAGGAATATTCATATCCTATGGATG-3’ (forward) and 5’-TTTAATTTTTAGTTTAAAGATGGTATC-3’ (reverse). After the double digestion with the restriction enzymes for the target genes and the plasmid (2 µl cutsmart buffer, 1 µl ndeI, 1 µl xhoI, 1000 ng template, ad 20 µl H_2_O), as well as the dephosphorylation of the plasmid (1 µl FastAp additional to the double digest mixture) for 2 h at 37 °C and the deactivation for 20 min at 65 °C, the Monarch® PCR & DNA Cleanup Kit (5 µg) (New England BioLabs® Inc., Ipswich Massachusetts) was used to purify the plasmid. In the following, the ligation product with T4-Ligase was transferred into *E. coli* DH5α by heat shock and sequenced with T7-promoter and T7-terminator primers after growing in LB-medium with 100 µg*ml^−1^ ampicillin. Plasmids of successful clones were extracted with the Presto™ Mini Plasmid Kit (Geneaid, New Taipei City Taiwan R.O.C.) and retransformed by heat shock in the expression strain *E. coli* BL21.

The protein expression was initiated by inoculating auto-induction medium ZYM5052^95^ including 100 µg*ml^−1^ ampicillin with the modified *E. coli* BL21 strain at 37 °C until the bacterial growth reached an OD_600nm_ of 0.6 in the media followed by the expression phase at 22 °C overnight. The bacterial cells were disrupted by sonification in the Hielscher Ultrasonics GmbH UP200S (3 × 1 min, amplitude 70%, cycle 0.5), and the enzymes were purified with the QIAGEN® Ni–NTA Fast Start Kit (Venlo, Netherlands). The purified enzyme was rebuffered in 0.1 M potassium phosphate Buffer (PPB, pH 7) until further use.

### Sequence and structural alignments of GH29 candidates

Protein structure predictions were calculated with AlphaFold2 and displayed with UCSF Chimera v.1.16, which was also used to generate the supplemental movie files^96^. Furthermore, extended structure comparisons on the protein database (PDB)^67^ were carried out with the Dali server^97^. First, similar protein structures were found through the PDB search tool, followed by an ‘All against All’ analysis, including the implemented enzymes and protein structures with a cut-off of the RMSD of 2.5 Â.

### GH29 α-L-fucosidase activity assay

Two GH29 enzymes (FUJM18, FUJM20) were tested with a 4-Nitrophenyl-α-L-fucopyranoside assay. The enzyme activity was conducted in triplicates at temperatures 22 °C, 28 °C, 37 °C, 50 °C, 60 °C, 70 °C, and 80 °C and from pH values between 4 and 8 (pH 4: sodium acetate; pH 5–6: sodium citrate; pH 7–8: HEPES). For this, 180 µl 0.1 M reaction buffer was mixed with 10 µl of 0.15 mg*ml^−1^ enzyme PBB mixture. After 20 min of incubation at the assay temperature, 10 µl of 10 mM *p*NP-α-L-Fuc diluted in Aqua bidest were added and incubated for 15 min. 20 µl 2 M sodium carbonate was used to adjust the pH to the same level before measuring the absorbance at 405 nm with the plate reader Synergy H1 (BioTek, Winooski). A standard curve using 4-nitrophenol was measured to calculate enzyme activity units (U) in mmol*min^−1^ and enzyme activity rates.

### Supplementary Information

Supplementary Video 1.

Supplementary Video 2.

Supplementary Video 3.

Supplementary Information 1.

## Data Availability

For the sequences’ functional characterisation, the Integrated Microbial Genomes (IMG) pipeline and homology searches were used^79,98^. Sequence data for the 16S rRNA analyses have been submitted to the European Nucleotide Archive. They are publicly available under accession no. PRJEB65586 and under the IMG ID Ga0502370; Ga0502371; Ga0502372; and Ga0502373.
