# Supplementary material for: Community dynamics and metagenomic analyses reveal Bacteroidota's role in widespread enzymatic Fucus vesiculosus cell wall degradation
Source: Sci Rep. 2024 May 3;14:10237. doi: 10.1038/s41598-024-60978-8 (PMC11068906; doi:10.1038/s41598-024-60978-8)
Supplement: Supplementary file 4 — Supplementary Information 1. [file 41598_2024_60978_MOESM4_ESM.pdf]

Community dynamics and metagenomic analyses reveal *Bacteroidota's* role in widespread enzymatic *Fucus vesiculosus* cell wall degradation

Jascha F.H. Macdonald[1], Pablo Pérez-García[1,2], Yannik K.-H. Schneider[3], Patrick Blümke[4], Daniela Indenbirken[4], Jeanette H. Andersen[3], Ines Krohn[1]*, and Wolfgang R. Streit[1]

[1] University of Hamburg, Biocenter Klein Flottbek, Department of Microbiology and Biotechnology, Hamburg, Germany

[2] Kiel University, Institute for General Microbiology, Molecular Microbiology, Kiel, Germany

[3] Marbio, Faculty of Biosciences, Fisheries and Economics, UiT—The Arctic University of Norway, Tromsø, Norway

[4] Leibniz Institute of Virology, Technology Platform Next Generation Sequencing, Hamburg, Germany

Keywords: *Fucus vesiculosus,* Cell wall degradation, *Bacteroidota*, α-L-fucosidases

***For Correspondence**

E-Mail: ines.krohn@uni-hamburg.de

Department of Microbiology and Biotechnology,

Institute of Plant science and Microbiology, University of Hamburg,

Ohnhorststr.18,

D-22609 Hamburg, Germany,

Tel. (+49) 40-42816-521

Fax. (+49) 40-42816-459

Supplementary Table 1

Numerical data referring to Figure 2. R1-R3: replicate, $OD_{600\ nm}$: $OD_{600\ nm}$ optical density of the enrichment culture. PHAH $OD_{410\ nm}$: determined absorbance at 410 nm of para-hydroxybenzoic acid hydrazide assay. PHAHFuc: converted PHAH $OD_{410\ nm}$ resemble L-fucose concentration calculated on a standard curve of y = 0.0448x+0.0052 (n=21) determined by 7 concentrations of L-(-)-fucose in technical triplicates

DAY	$OD_{600\ nm}$ R1	$OD_{600\ nm}$ R2	$OD_{600\ nm}$ R3	PHAH $OD_{410\ nm}$ R1	PHAH $OD_{410\ nm}$ R2	PHAH $OD_{410\ nm}$ R3	PHAHFUC [MOL] R1	PHAHFUC [MOL] R2	PHAHFUC [MOL] R3
d0	0,102	0,1025	0,1077	0,006	0,008	0,007	0,017857143	0,0625	0,040178571
d1	0,1242	0,1206	0,1399	0,007	0,013	0,017	0,040178571	0,174107143	0,263392857
d2	0,331	0,3344	0,312	0,026	0,021	0,029	0,464285714	0,352678571	0,53125
d3	0,4432	0,4775	0,4529	0,063	0,064	0,079	1,290178571	1,3125	1,647321429
d4	0,6983	0,7132	0,7362	0,089	0,094	0,121	1,870535714	1,982142857	2,584821429
d5	0,915	0,9105	0,9227	0,103	0,158	0,169	2,183035714	3,410714286	3,65625
d6	1,0334	1,0234	1,017	0,164	0,203	0,198	3,544642857	4,415178571	4,303571429
d7	1,0784	1,0998	1,0899	0,141	0,113	0,113	3,03125	2,40625	2,40625
d8	1,0962	1,1203	1,1151	0,089	0,078	0,085	1,870535714	1,625	1,78125
d9	1,0879	1,1123	1,1057	0,093	0,101	0,111	1,959821429	2,138392857	2,361607143
d10	1,1563	1,1511	1,1195	0,076	0,086	0,102	1,580357143	1,803571429	2,160714286
d11	1,203	1,1498	1,1494	0,079	0,087	0,104	1,647321429	1,825892857	2,205357143
d12	1,066	1,1087	1,0685	0,074	0,058	0,069	1,535714286	1,178571429	1,424107143
d13	1,1751	1,1314	1,1592	0,064	0,071	0,079	1,3125	1,46875	1,647321429
d14	0,8487	0,8945	0,879	0,056	0,056	0,058	1,133928571	1,133928571	1,178571429
d15	0,9871	0,9581	0,9855	0,055	0,057	0,061	1,111607143	1,15625	1,245535714
d16	0,9925	0,8165	0,7656	0,074	0,06	0,074	1,535714286	1,223214286	1,535714286

Supplementary Table 2
IMG-accession of provided metagenomes of *F. vesiculosus* microbiomes

IMG/MER ACCESSION NUMBER	NUMBER OF CONTIGS	GC CONTENT	LONGEST CONTIG	MEAN CONTIG	TOTAL CONTIG LENGTH
**Ga0502370**	399,970	45.29%	211.39 Kbp	707.96 bp	283.16 Mbp
**Ga0502371**	1,143,468	45.45%	216.74 Kbp	640.13 bp	731.97 Mbp
**Ga0502372**	715,725	45.23%	350.84 Kbp	695.60 bp	497.86 Mbp
**Ga0502373**	1,073,390	44.22%	283.36 Kbp	578.47 bp	620.93 Mbp

Supplementary Table 3
Numerical data referring to Figure 6. Enzyme unit (U) in mmol*min^{-1}. Enzyme activities were measured in triplicates and calculated on a standard curve y = 2.6702x+0.0529 (n= 18) determined by 6 concentrations of 4-nitrophenol in triplicates.

GEN	PH	TEMPERATURE	ENZYME ACTIVITY [U/MG]	STANDART DEVIATION
**FUJM18**	pH 6	22 °C	7.419	0.765
FUJM18	pH 6	28 °C	10.565	7.759
FUJM18	pH 4	37 °C	11.497	1.655
FUJM18	pH 5	37 °C	13.289	4.860
FUJM18	pH 6	37 °C	37.391	16.692
FUJM18	pH 7	37 °C	15.281	6.285
FUJM18	pH 8	37 °C	8.784	4.641
FUJM18	pH 6	50 °C	33.379	4.438
FUJM18	pH 6	60 °C	41.702	7.018
FUJM18	pH 6	70 °C	32.736	11.205
FUJM18	pH 4	80 °C	0	0
FUJM18	pH 5	80 °C	0	0
FUJM18	pH 6	80 °C	61.176	12.790
FUJM18	pH 7	80 °C	4.113	1.237
FUJM18	pH 8	80 °C	0	0

FUJM20	pH 6	22 °C	8.806	0.838
FUJM20	pH 6	28 °C	8.773	4.434
FUJM20	pH 4	37 °C	12.485	0.953
FUJM20	pH 5	37 °C	8.568	1.265
FUJM20	pH 6	37 °C	29.385	15.242
FUJM20	pH 7	37 °C	2.709	0.510
FUJM20	pH 8	37 °C	1.512	2.036
FUJM20	pH 6	50 °C	28.053	8.299
FUJM20	pH 6	60 °C	33.490	5.430
FUJM20	pH 6	70 °C	39.427	5.309
FUJM20	pH 4	80 °C	0	0
FUJM20	pH 5	80 °C	11.020	6.731
FUJM20	pH 6	80 °C	41.768	1.346
FUJM20	pH 7	80 °C	31.338	2.282
FUJM20	pH 8	80 °C	0	0
CONTROL	pH 6	22 °C	0	0
CONTROL	pH 6	28 °C	0	0
CONTROL	pH 4	37 °C	0	0
CONTROL	pH 5	37 °C	0	0
CONTROL	pH 6	37 °C	0	0
CONTROL	pH 7	37 °C	0	0
CONTROL	pH 8	37 °C	0	0
CONTROL	pH 6	50 °C	0	0
CONTROL	pH 6	60 °C	0	0
CONTROL	pH 6	70 °C	0	0
CONTROL	pH 4	80 °C	0	0
CONTROL	pH 5	80 °C	0	0
CONTROL	pH 6	80 °C	0	0
CONTROL	pH 7	80 °C	0	0
CONTROL	pH 8	80 °C	0	0

Supplementary Table 4
Enzyme classes and families involved in algae carbohydrate degradation

ALGAE CELL WALL POLYMERE	ALGAE	GH FAMILIES	PL FAMILIES	SULFATASE FAMILIES	CBM FAMILIES	CE FAMILIES
**FUCOIDAN**	Phaeophyceae	1, 29, 30, 35, 92, 95, 107, 139, 141, 149, 151, 168	-	1_15, 1_16, 1_17, 1_25	35, 47, 51	-
**LAMINARIN**	Phaeophyceae/ Bacillariophyta	3, 16, 17, 30, 55, 81	-	-	4, 6, 32, 54, 56	-
**ALGINATE**	Phaeophyceae	-	5, 6, 7, 14, 15, 17, 18, 31, 32, 34, 36, 38, 41	-	2, 13, 16, 32, 35	-
**CELLULOSE**	Phaeophyceae/ Chlorophyta	1, 3, 5, 6, 7, 8, 9, 10, 12, 16, 26, 39, 44, 45, 48, 51, 55, 74	-	-	1, 2, 3, 4, 5, 6, 9, 10, 17, 22, 28, 32, 37, 46, 60, 63, 65, 72, 76, 78, 80, 81	4
**CARRAGEENAN**	Rhodophyta	16, 82, 150, 164	-	-	16, 92	-
**MANNAN**	Chlorophyta/ Rhodophyta	1, 2, 5, 26, 38, 44, 45, 47, 76, 92, 99, 113, 125, 130, 134	-	-	1, 2, 3, 5, 6, 10, 16, 23, 27, 32, 35, 59	-
**XYLAN**	Chlorophyta/ Rhodophyta	3, 5, 8, 10, 11, 26, 30, 43, 44, 62, 141	-	-	1, 2, 3, 4, 5, 6, 9, 10, 13, 15, 22, 31, 35, 36, 37, 48, 59, 60, 64, 72, 86	1, 3, 4, 20
**AGAR**	Rhodophyta	16, 50, 86, 96, 117, 118	-	-	6, 13	-
**PORPHYRAN**	Rhodophyta	16, 86	-	-	13, 92	-

**PECTIN**	Chlorophyta/ Bacillariophyta	1, 2, 5, 16, 28, 30, 35, 42, 43, 51, 53, 54, 62	1, 2, 3, 4, 9, 11, 26	-	1, 13	1, 8, 12
**ULVAN**	Chlorophyta	2, 3, 39, 43, 78, 88, 92, 105	24, 25, 28, 40	-	-	90

Supplementary Table 5
16S-Amplicon Primer for library preparation

SAMPLE	NAME	FUNCTION	SEQUENCE	NO OF READS
	515f	16S-Amplicon forward	AATGATACGGCGACCACCGAGATCTACACTATGGTA ATTGTGTGCCAGCMGCCGCGGTAA	
d0	806rcbc11	16S-Amplicon reverse	CAAGCAGAAGACGGCATACGAGATAATTGTGTCGGA AGTCAGTCAGCCGGACTACHVGGGTWTCTAAT	318,500
d3	806rcbc12	16S-Amplicon reverse	CAAGCAGAAGACGGCATACGAGATTGCATACACTGG AGTCAGTCAGCCGGACTACHVGGGTWTCTAAT	96,165
d6	806rcbc13	16S-Amplicon reverse	CAAGCAGAAGACGGCATACGAGATAGTCGAACGAG GAGTCAGTCAGCCGGACTACHVGGGTWTCTAAT	87,960
d9	806rcbc14	16S-Amplicon reverse	CAAGCAGAAGACGGCATACGAGATACCAGTGACTCA AGTCAGTCAGCCGGACTACHVGGGTWTCTAAT	207,850
d12	806rcbc15	16S-Amplicon reverse	CAAGCAGAAGACGGCATACGAGATGAATACCAAGTC AGTCAGTCAGCCGGACTACHVGGGTWTCTAAT	50,994
d15	806rcbc16	16S-Amplicon reverse	CAAGCAGAAGACGGCATACGAGATGTAGATCGTGTA AGTCAGTCAGCCGGACTACHVGGGTWTCTAAT	65,269

Supplementary Table 6
Hidden Markov Models for Glycosyl Hydrolase (GH) and Sulfatases (S1)

CATEGORY	FAMILY	ACCESSION	TYPE	DESCRIPTION	SOURCE
GH	1	PF00232	Domain	Glycosyl hydrolase family 1	Pfam
GH	2	PF00703	Domain	Glycosyl hydrolases family 2	Pfam
GH	2_C	PF02836	Domain	Glycosyl hydrolases family 2, TIM barrel domain	Pfam
GH	2_N	PF02837	Domain	Glycosyl hydrolases family 2, sugar binding domain	Pfam
GH	3	PF00933	Domain	Glycosyl hydrolase family 3 N terminal domain	Pfam
GH	3_C	PF01915	Domain	Glycosyl hydrolase family 3 C-terminal domain	Pfam
GH	4	PF02056	Family	Family 4 glycosyl hydrolase	Pfam
GH	4C	PF11975	Domain	Family 4 glycosyl hydrolase C-terminal domain	Pfam
GH	5	PF14872	Family	Hypothetical glycoside hydrolase 5	Pfam
GH	5_C	PF18564	Domain	Glycoside hydrolase family 5 C-terminal domain	Pfam
GH	6	PF01341	Domain	Glycosyl hydrolases family 6	Pfam
GH	6_2	PF14871	Family	Hypothetical glycosyl hydrolase 6	Pfam
GH	7	PF00840	Domain	Glycosyl hydrolase family 7	Pfam
GH	8	PF01270	Repeat	Glycosyl hydrolases family 8	Pfam
GH	9	PF00759	Repeat	Glycosyl hydrolase family 9	Pfam
GH	10	PF00331	Domain	Glycosyl hydrolase family 10	Pfam
GH	11	PF00457	Domain	Glycosyl hydrolases family 11	Pfam
GH	12	PF01670	Domain	Glycosyl hydrolase family 12	Pfam
GH	13	PF14883	Family	Hypothetical glycosyl hydrolase family 13	Pfam
GH	14	PF01373	Domain	Glycosyl hydrolase family 14	Pfam
GH	15	PF00723	Repeat	Glycosyl hydrolases family 15	Pfam
GH	16	PF00722	Domain	Glycosyl hydrolases family 16	Pfam
GH	17	PF00332	Domain	Glycosyl hydrolases family 17	Pfam
GH	18	PF00704	Domain	Glycosyl hydrolases family 18	Pfam
GH	19	PF00182	Domain	Chitinase class I	Pfam
GH	20	PF00728	Domain	Glycosyl hydrolase family 20, catalytic domain	Pfam

GH	20b	PF02838	Domain	Glycosyl hydrolase family 20, domain 2	Pfam
GH	25	PF01183	Domain	Glycosyl hydrolases family 25	Pfam
GH	26	PF02156	Domain	Glycosyl hydrolase family 26	Pfam
GH	28	PF00295	Repeat	Glycosyl hydrolases family 28	Pfam
GH	29	PF01120	Family	Alpha-L-fucosidase	Pfam
GH	30	PF02055	Domain	Glycosyl hydrolase family 30 TIM-barrel domain	Pfam
GH	30_2	PF14587	Domain	O-Glycosyl hydrolase family 30	Pfam
GH	30C	PF17189	Domain	Glycosyl hydrolase family 30 beta sandwich domain	Pfam
GH	31	PF01055	Family	Glycosyl hydrolases family 31	Pfam
GH	32C	PF08244	Domain	Glycosyl hydrolases family 32 C terminal	Pfam
GH	32N	PF00251	Domain	Glycosyl hydrolases family 32 N-terminal domain	Pfam
GH	35	PF01301	Domain	Glycosyl hydrolases family 35	Pfam
GH	36	PF17167	Repeat	Glycosyl hydrolase 36 superfamily, catalytic domain	Pfam
GH	36C	PF16874	Domain	Glycosyl hydrolase family 36 C-terminal domain	Pfam
GH	36N	PF16875	Domain	Glycosyl hydrolase family 36 N-terminal domain	Pfam
GH	38	PF18438	Domain	Glycosyl hydrolases family 38 C-terminal domain 1	Pfam
GH	38C	PF07748	Domain	Glycosyl hydrolases family 38 C-terminal domain	Pfam
GH	38N	PF01074	Domain	Glycosyl hydrolases family 38 N-terminal domain	Pfam
GH	39	PF01229	Family	Glycosyl hydrolases family 39	Pfam
GH	42	PF02449	Domain	Beta-galactosidase	Pfam
GH	42C	PF08533	Domain	Beta-galactosidase C-terminal domain	Pfam
GH	42M	PF08532	Domain	Beta-galactosidase trimerisation domain	Pfam
GH	43	PF04616	Family	Glycosyl hydrolases family 43	Pfam
GH	44	PF12891	Domain	Glycoside hydrolase family 44	Pfam
GH	45	PF02015	Domain	Glycosyl hydrolase family 45	Pfam
GH	46	PF01374	Domain	Glycosyl hydrolase family 46	Pfam
GH	47	PF01532	Repeat	Glycosyl hydrolase family 47	Pfam

GH	48	PF02011	Repeat	Glycosyl hydrolase family 48	Pfam
GH	49	PF03718	Repeat	Glycosyl hydrolase family 49	Pfam
GH	49N	PF17433	Domain	Glycosyl hydrolase family 49 N-terminal Ig-like domain	Pfam
GH	52	PF03512	Family	Glycosyl hydrolase family 52	Pfam
GH	53	PF07745	Domain	Glycosyl hydrolase family 53	Pfam
GH	56	PF01630	Domain	Hyaluronidase	Pfam
GH	57	PF03065	Domain	Glycosyl hydrolase family 57	Pfam
GH	59	PF02057	Family	Glycosyl hydrolase family 59	Pfam
GH	59M	PF17387	Domain	Glycosyl hydrolase family 59 central domain	Pfam
GH	62	PF03664	Family	Glycosyl hydrolase family 62	Pfam
GH	63	PF03200	Repeat	Glycosyl hydrolase family 63 C-terminal domain	Pfam
GH	63N	PF16923	Domain	Glycosyl hydrolase family 63 N-terminal domain	Pfam
GH	64	PF16483	Domain	Beta-1,3-glucanase	Pfam
GH	65C	PF03633	Family	Glycosyl hydrolase family 65, C-terminal domain	Pfam
GH	65m	PF03632	Repeat	Glycosyl hydrolase family 65 central catalytic domain	Pfam
GH	65N	PF03636	Family	Glycosyl hydrolase family 65, N-terminal domain	Pfam
GH	65N_2	PF14498	Domain	Glycosyl hydrolase family 65, N-terminal domain	Pfam
GH	66	PF13199	Domain	Glycosyl hydrolase family 66	Pfam
GH	67C	PF07477	Domain	Glycosyl hydrolase family 67 C-terminus	Pfam
GH	67M	PF07488	Domain	Glycosyl hydrolase family 67 middle domain	Pfam
GH	67N	PF03648	Domain	Glycosyl hydrolase family 67 N-terminus	Pfam
GH	68	PF02435	Family	Levansucrase/Invertase	Pfam
GH	70	PF02324	Family	Glycosyl hydrolase family 70	Pfam
GH	71	PF03659	Family	Glycosyl hydrolase family 71	Pfam
GH	72	PF03198	Domain	Glucanosyltransferase	Pfam
GH	75	PF07335	Family	Fungal chitosanase of glycosyl hydrolase group 75	Pfam
GH	76	PF03663	Repeat	Glycosyl hydrolase family 76	Pfam

GH	77	PF02446	Domain	4-alpha-glucanotransferase	Pfam
GH	79C	PF16862	Domain	Glycosyl hydrolase family 79 C-terminal beta domain	Pfam
GH	79n	PF03662	Domain	Glycosyl hydrolase family 79, N-terminal domain	Pfam
GH	80	PF13647	Domain	Glycosyl hydrolase family 80 of chitosanase A	Pfam
GH	81	PF03639	Domain	Glycosyl hydrolase family 81 N-terminal domain	Pfam
GH	85	PF03644	Family	Glycosyl hydrolase family 85	Pfam
GH	88	PF07470	Repeat	Glycosyl Hydrolase Family 88	Pfam
GH	92	PF07971	Repeat	Glycosyl hydrolase family 92	Pfam
GH	92N	PF17678	Domain	Glycosyl hydrolase family 92 N-terminal domain	Pfam
GH	97	PF10566	Domain	Glycoside hydrolase 97	Pfam
GH	98C	PF08307	Domain	Glycosyl hydrolase family 98 C-terminal domain	Pfam
GH	98M	PF08306	Domain	Glycosyl hydrolase family 98	Pfam
GH	99	PF16317	Domain	Glycosyl hydrolase family 99	Pfam
GH	100	PF12899	Repeat	Alkaline and neutral invertase	Pfam
GH	101	PF12905	Domain	Endo-alpha-N-acetylgalactosaminidase	Pfam
GH	101C	PF17451	Domain	Glycosyl hydrolase 101 beta sandwich domain	Pfam
GH	106	PF17132	Family	alpha-L-rhamnosidase	Pfam
GH	107	-	Family	Glycosyl hydrolase family 107	caZy/This study
GH	108	PF05838	Domain	Glycosyl hydrolase 108	Pfam
GH	114	PF03537	Domain	Glycoside-hydrolase family GH114	Pfam
GH	115	PF15979	Family	Glycosyl hydrolase family 115	Pfam
GH	116N	PF12215	Family	beta-glucosidase 2, glycosyl-hydrolase family 116 N-term	Pfam
GH	125	PF06824	Repeat	Metal-independent alpha-mannosidase (GH125)	Pfam
GH	127	PF07944	Repeat	Beta-L-arabinofuranosidase, GH127	Pfam
GH	129	PF11308	Family	Glycosyl hydrolases related to GH101 family, GH129	Pfam
GH	130	PF04041	Family	beta-1,4-mannooligosaccharide phosphorylase	Pfam

GH	168	-	Family	Glycosyl hydrolase family 168	caZy/This study
Sulfatase	S1_17	-	Family	subfamily S1_17	SulfAtlas
Sulfatase	S1_25	-	Family	subfamily S1_25	SulfAtlas

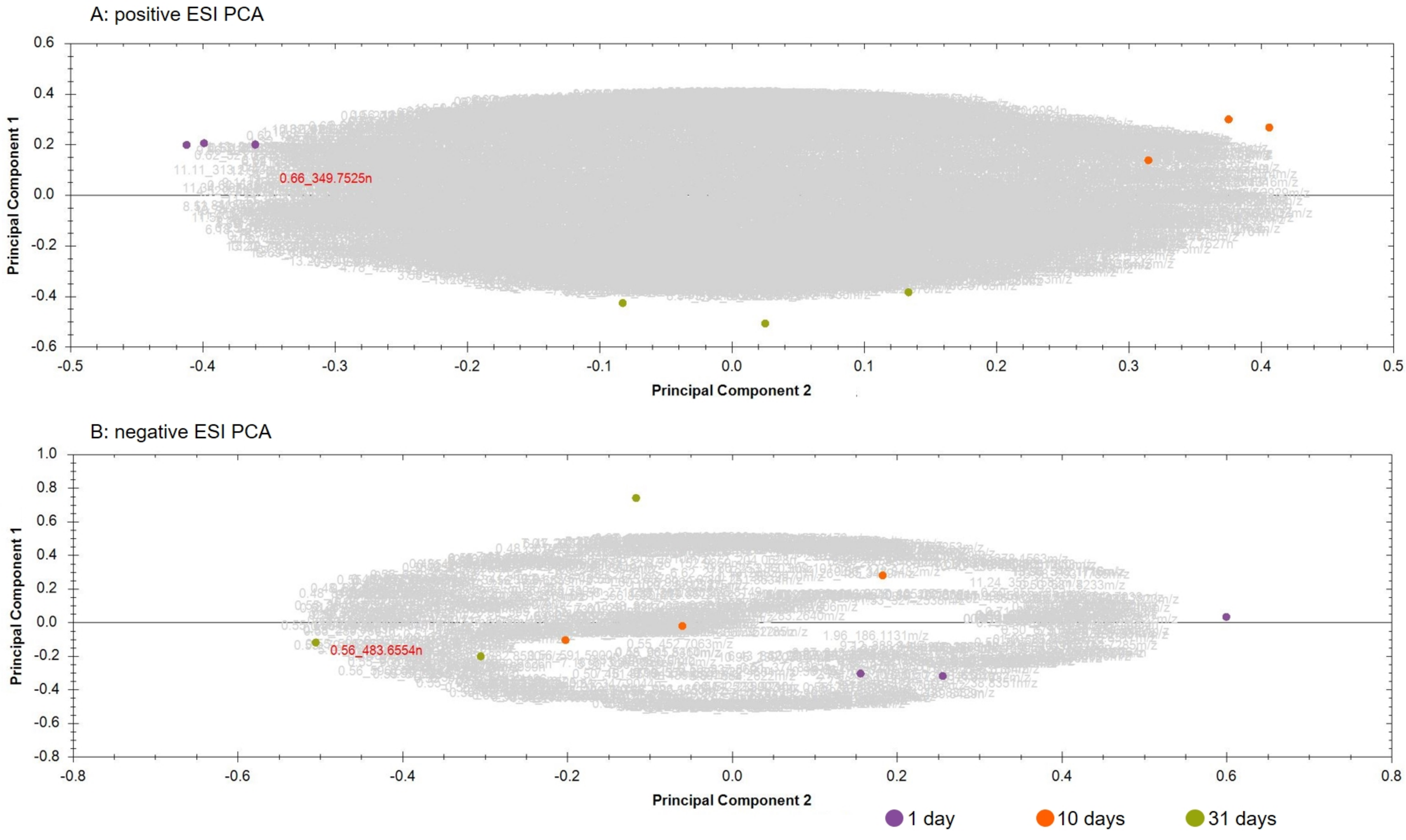

Supplemental Figure 1: Principal component analysis (PCA) of the features for MS-datasets recorded using A: positive Electrospray (ESI); and B: negative ESI. For each time point (1, 10 and 31 days) three replicates were incubated and analysed.

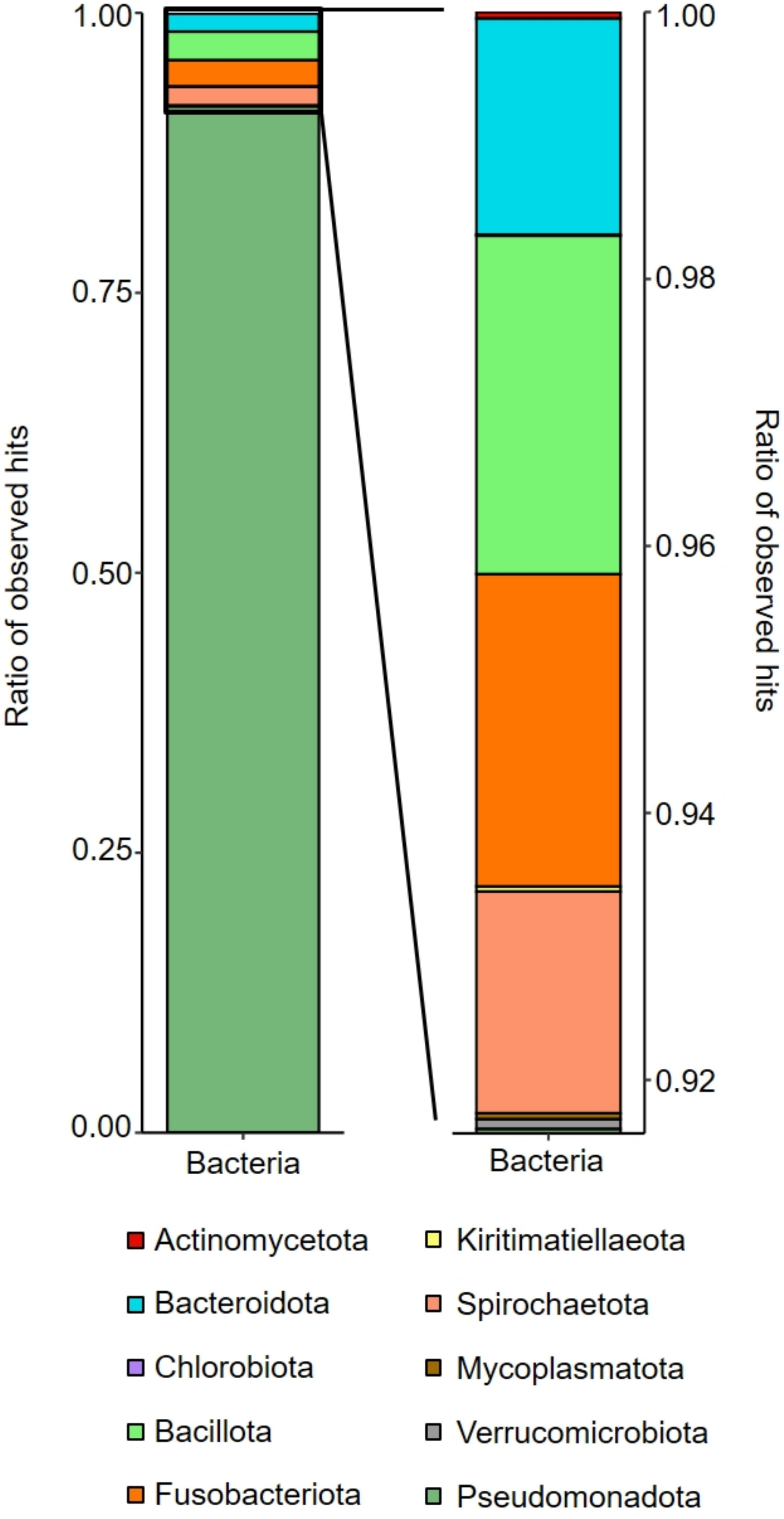

Supplementary Figure 2: Diversity and phylogeny of *F. vesiculosus* microbiome in enrichment cultures after 9 days. Mean metagenome bacterial phylogeny of 4 enrichment cultures representing phyla with a ratio of all covered genes higher than 0.0001% within the whole metagenome. Standard deviation is equal to or below 0.01 for every phyla besides Proteobacteria with ± 0.025.

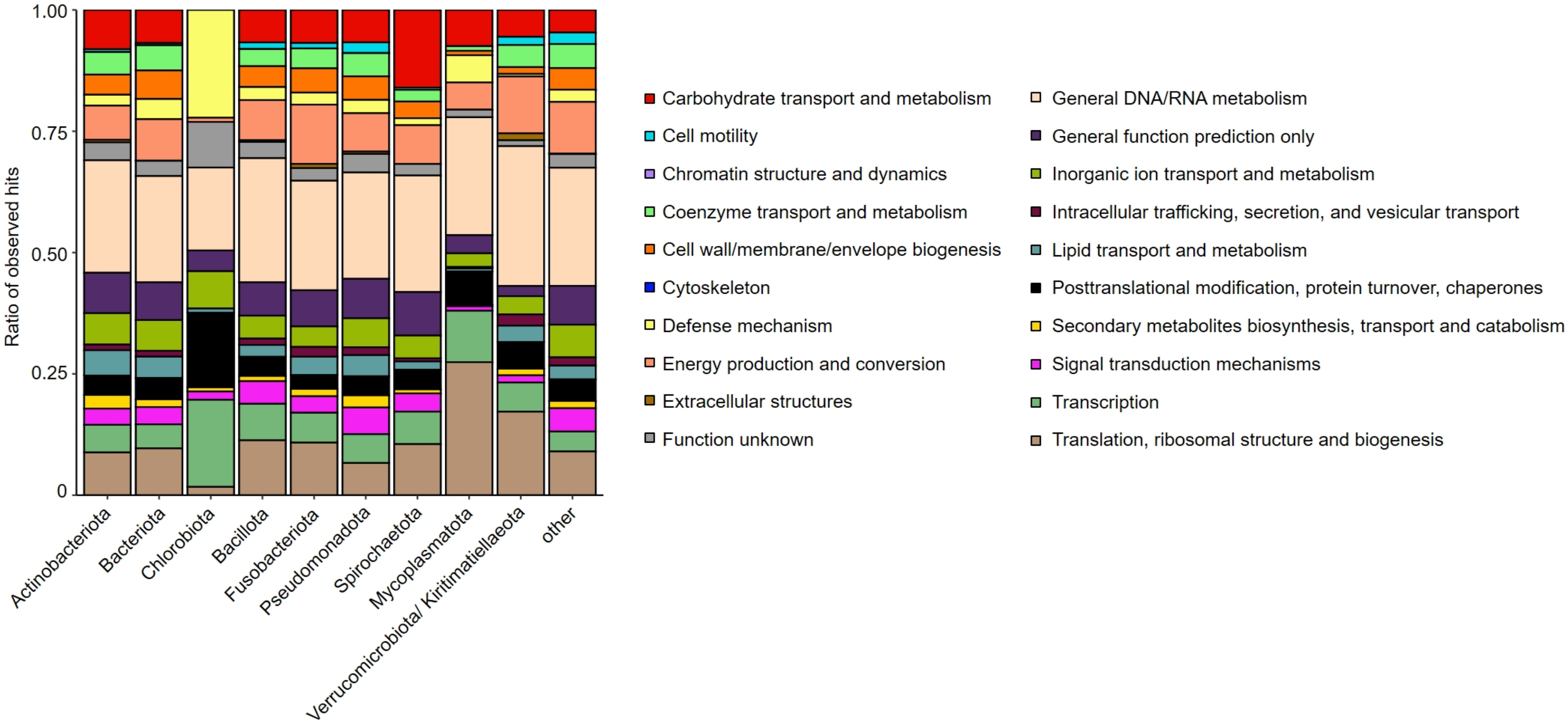

Supplementary Figure 3: Clusters of orthologous groups of proteins of bacteria with a ratio of all covered genes higher than 0.0001% within the whole metagenome in *Fucus vesiculosus* enrichment cultures after 9 days.

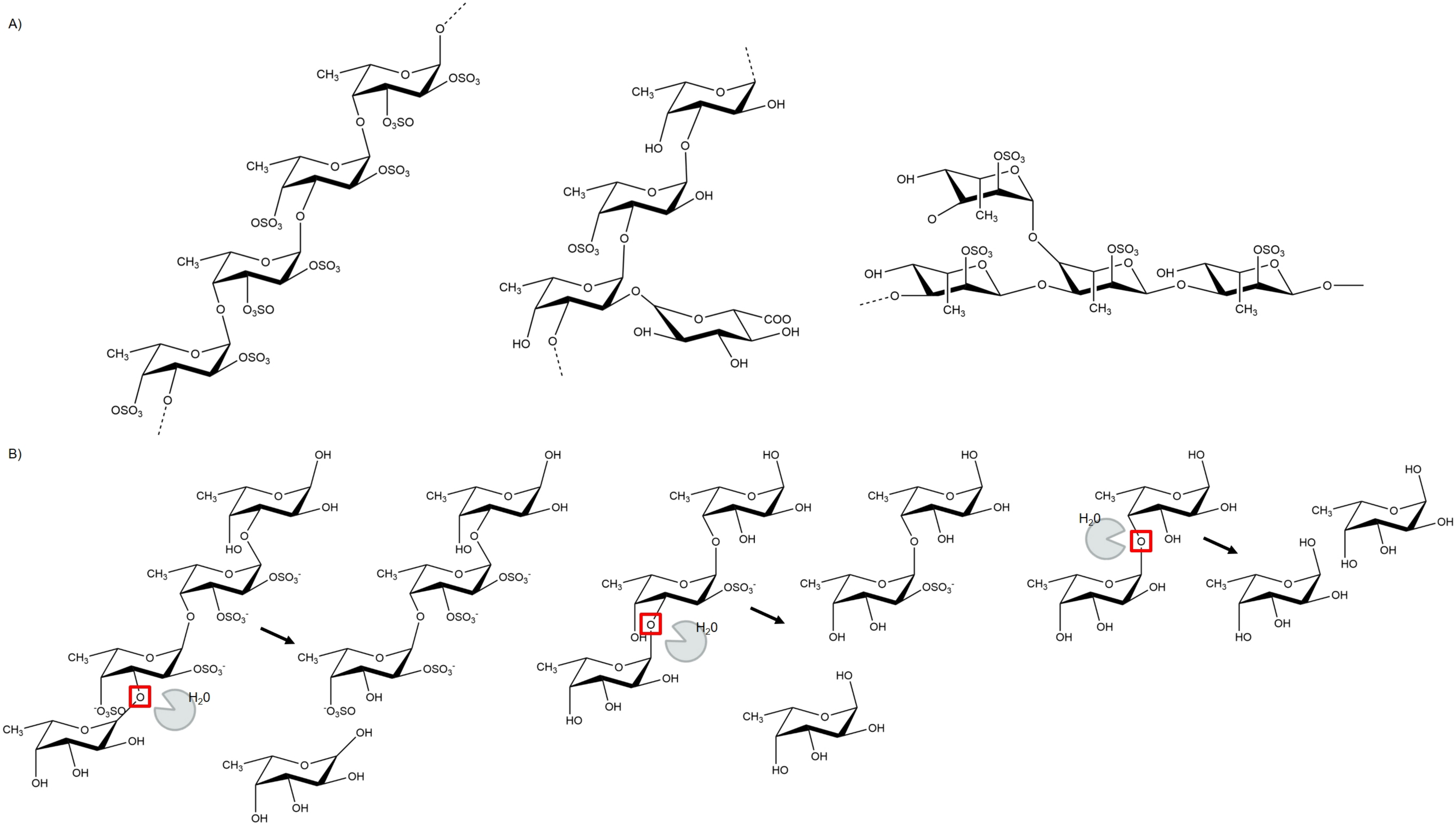

Supplementary Figure 4: Detailed schematic structure and function of GH29 enzymes. A) Examples of sulphated fucoidan structures. Homofucan as backbone structure and heterofucan with crosslinks. B) Fucoidan degradation catalyse with GH29. The structure of homofucan with sulphated α 1,3/1,4 linked L-fucose is degraded in multiple steps. Presented are enzymatic reactions with GH29 α L fucosidase. Structures generated with ChemDraw v.21.0.0. Adapted from Li et al. (2022) 49.

Supplemental Movie 1: Structure comparison of predicted FUJM18 protein structure and α-L-fucosidase isoenzyme 1 from *Paenibacillus thiaminolyticus* (6GN6) from the PDB database. Grey: α-L-fucosidase isoenzyme 1 from *Paenibacillus thiaminolyticus.* Orange: JUFM18 structure prediction by Alphafold2. BLUE: α-L-fucosidase isoenzyme 1 from *Paenibacillus thiaminolyticus* Chain D.

Supplemental Movie 2: Structure comparison of predicted FUJM20 protein structure and α-L-fucosidase isoenzyme 1 from *Paenibacillus thiaminolyticus* (6GN6) from the PDB database. Grey: α-L-fucosidase isoenzyme 1 from *Paenibacillus thiaminolyticus.* Green: JUFM20 structure prediction by Alphafold2. BLUE: α-L-fucosidase isoenzyme 1 from *Paenibacillus thiaminolyticus* Chain D.

Supplemental Movie 3: Structure comparison of predicted FUJM18 and predicted FUJM20. Orange: JUFM18 structure prediction by Alphafold2. Green: JUFM20 structure prediction by Alphafold2.
